# Hand Preference in Stuttering: Meta-Analyses

**DOI:** 10.1007/s11065-023-09617-z

**Published:** 2023-10-05

**Authors:** Marietta Papadatou-Pastou, Anastasia-Konstantina Papadopoulou, Christos Samsouris, Annakarina Mundorf, Maria-Myrto Valtou, Sebastian Ocklenburg

**Affiliations:** 1 National and Kapodistrian University of Athens, Athens, Greece; 2 Biomedical Research Foundation, Academy of Athens, Athens, Greece; 3 Institute for Systems Medicine and Department of Human Medicine, MSH Medical School Hamburg, Hamburg, Germany; 4 Department of Psychology, Medical School Hamburg, Hamburg, Germany; 5 ICAN Institute for Cognitive and Affective Neuroscience, Medical School Hamburg, Hamburg, Germany; 6 Institute of Cognitive Neuroscience, Biopsychology, Department of Psychology, Ruhr-University Bochum, Bochum, Germany

**Keywords:** Handedness, Stuttering, Stammering, Hand preference, Meta-analysis

## Abstract

Reduced hemispheric asymmetries, as well as their behavioral manifestation in the form of atypical handedness (i.e., non-right, left-, or mixed-handedness), are linked to neurodevelopmental disorders, such as autism spectrum disorder, and several psychiatric disorders, such as schizophrenia. One neurodevelopmental disorder that is associated with reduced hemispheric asymmetries, but for which findings on behavioral laterality are conflicting, is stuttering. Here, we report a series of meta-analyses of studies that report handedness (assessed as hand preference) levels in individuals who stutter (otherwise healthy) compared to controls. For this purpose, articles were identified via a search in PubMed, Scopus, and PsycInfo (13 June 2023). On the basis of *k* = 52 identified studies totaling *n* = 2590 individuals who stutter and *n* = 17,148 controls, five random effects meta-analyses were conducted: four using the odds ratio [left-handers (forced choice); left-handers (extreme); mixed-handers; non-right-handers vs. total)] and one using the standardized difference in means as the effect size. We did not find evidence of a left (extreme)- or mixed-handedness difference or a difference in mean handedness scores, but evidence did emerge, when it came to left-handedness (forced-choice) and (inconclusively for) non-right-handedness. Risk-of-bias analysis was not deemed necessary in the context of these meta-analyses. Differences in hand skill or strength of handedness could not be assessed as no pertinent studies were located. Severity of stuttering could not be used s a moderator, as too few studies broke down their data according to severity. Our findings do not allow for firm conclusions to be drawn on whether stuttering is associated with reduced hemispheric asymmetries, at least when it comes to their behavioral manifestation.

**Supplementary Information:**

The online version contains supplementary material available at 10.1007/s11065-023-09617-z.

## Hand Preference in Stuttering: Meta-Analyses

Individuals diagnosed with neurodevelopmental disorders, such as autism spectrum disorder, or several psychiatric disorders, show reduced hemispheric asymmetries and also a higher prevalence of atypical (i.e., non-right-, left- or mixed-) handedness than the general population (Mundorf & Ocklenburg, 2021; Mundorf et al., 2021). One neurodevelopmental disorder that is associated with altered functional hemispheric asymmetries in the language domain but shows rather ambiguous empirical results on the behavioral level (i.e., handedness), is stuttering (Brosch et al., 1999; Cavenagh et al., 2015; Mohammadi & Papadatou-Pastou, 2020; Mundorf & Ocklenburg, 2021; Mundorf et al., 2021; Vreeswijk et al., 2019). The World Health Organisation defines stuttering as (2010, para. F98.5): “speech that is characterized by the frequent repetitions or prolongation of sounds or syllables or words, or by frequent hesitations or pauses that disrupt the rhythmic flow of speech.” In the latest version of the Diagnostic and Statistical Manual of the American Psychiatric Association (DSM-5; American Psychiatric Association, 2013), “stuttering” is no longer an official diagnosis, with the name of the disorder having changed to Childhood-Onset Fluency Disorder (note: in the present manuscript we will adopt the term “stuttering,” as a number of studies that have predated this change are discussed). Generally, around 1% of children and adolescents as well as 0.2% of women and 0.8% of men worldwide suffer from stuttering with high heritability estimates between 70 to 80%. Treatment onset is often delayed and, for most of the treatment options, only insufficient evidence for treatment success exists (Neumann et al., 2017).

Earlier studies on electroencephalogram (EEG) activity propose that the left language-dominant brain hemisphere is most active during speech and language tasks in fluent speakers, with atypical activation in stutterers (Büchel & Sommer, 2004; Moore & Haynes, 1980). Thus, stuttering, as a disorder of the brain (Sommer et al., 2002), is accompanied by atypically low function of left hemispheric speech areas. Interestingly, this lower left-hemispheric activity can be increased to comparable normal levels in induced fluency (e.g., with chorus reading) (Maguire et al., 2002). The notion of atypical hemispheric lateralization associated with difficulties in language processing and speech comes as no surprise as most individuals show stronger activations in the left hemisphere when processing language (Güntürkün et al., 2020; Hugdahl & Westerhausen, 2016). This left-hemispheric lateralization of language processing is also evident on a structural level. In a study in healthy participants, in-vivo imaging was used to quantify the microcircuitry in terms of axon and dendrite complexity of the left and right planum temporale, an important area for speech perception. The researchers found that a higher density of dendrites and axons in the left posterior temporal lobe was linked to faster neurophysiological processing of auditory speech (Ocklenburg et al., 2018). On the behavioral level, a link between language lateralization and handedness has been postulated (Ocklenburg et al., 2014a, b). This link may be due to shared ontogenetic factors but the strength of this correlation between handedness and language lateralization is dependent on the measures used to assess the two traits, that is on whether hand preference or hand skill is assessed (Ocklenburg et al., 2014a, 2014b). Others report that handedness, when measured by the pegboard test as a motor skill task, can predict of speech laterality, both in children and adults (Hodgson & Hudson, 2018). Interestingly, the researchers report that individuals with developmental motor coordination impairments also show atypical speech lateralization.

In 1947, Kypros Chrysanthis, one of the first scientists to investigate handedness in stuttering children, found a more than fourfold increase in left-handedness in children that stuttered (Chrysanthis, 1947). Thus, he proposed a link between atypical hemispheric lateralization and stuttering. Since then, several studies have examined a potential link between a left hand preference and stuttering. For example, in a population-based study with 446 children and adults that classify themselves as stutterers and controls, Records et al. (1977) did not find any differences in hand preference between females and males or stutterers and controls. However, they reported that in their study, both males and stutters tend to be less right-handed, reinforcing a connection between the two factors. In a prospective study including almost 80 stuttering children aged 3–9 years, Brosch et al. (1999) tested the children for hand preference and speech fluency at the study start and then 18 months later. They found that left-handed children had a poorer chance of attaining speech fluency when compared to right-handed stutterers. In a smaller study with children who stutter compared to children who speak fluently (*N* < 50 per group), parental-reported handedness was assessed. Interestingly, a significantly higher percentage of left-handed children who stutter (24.3%), with more boys being left-handed, compared to the control group (14.3%) was found (Cavenagh et al., 2015). Whillier and colleagues (2018) observed no difference in hand preference between stuttering adults (*N* < 20) and matched fluent speaking controls, with both groups favoring the right hand. In a sample of *N* < 20 adolescents and adults who stuttered and matched fluent speaking adults, Vreeswijk et al. (2019) found no difference in hand preference between the two groups. Another study investigating a link between the severity of stuttering and hand preference included data from 92 Kurdish children who stutter (23.90% females) and 91 control children (29.67% females). The analysis detected no differences in their hand preference, as reported by the parent-completed Edinburgh Handedness Inventory, and no correlation between the severity of stuttering and degree of handedness (Mohammadi & Papadatou-Pastou, 2020). Of note, it has also been proposed that stuttering is rather a consequence of forcing left-handers to write with their right hand (by confusing the existing hemispheric dominance) than a result of left-handedness itself (Kushner, 2011, 2012, 2017). One quite famous example for this hypothesis was England’s Duke of York, the future King George VI (1895–1952), who was naturally left-handed, had been forced to write with his right hand and started stuttering around the same time as he was forced to switch hands (Kushner, 2011). Since then, protests against retraining left-handers were supported by increasing scientific evidence and expert opinions (Kushner, 2011) that also lead to a greater understanding of the etiology of handedness (Kushner, 2017).

Given that most studies differ greatly in study design, sample size, and sample composition, these differences may lead to ambiguous results. Especially when investigating a neurodevelopmental disorder, such as stuttering, the age of the cohort included may have a great impact (e.g., when observing children versus adults). Moreover, differences in handedness phenotyping can also lead to contrasting findings (Ocklenburg et al., 2014a, 2014b; Paracchini, 2021). Thus, a meta-analysis of findings on handedness and stuttering is needed to disentangle the relation between handedness and stuttering and to control for confounders, such as small study bias and between-study heterogeneity. In recent years, handedness meta-analyses have been conducted for some neurodevelopmental disorders, such as attention deficit hyperactivity disorder (Nastou et al., 2022) and autism (Markou et al., 2017), and have significantly advanced the field of clinical neuroscience of lateralization by providing evidence of elevated levels of non-right-handedness for both disorders (albeit evidence was much stronger for autism).

Thus, the aim of the present study is to address handedness differences between individuals who stutter compared to control participants. Our main, pre-registered, questions are as follows:

1. Do individuals who stutter have elevated levels of left-handedness, mixed-handedness, and/or non-right-handedness compared to control participants?
2. Do other factors, such as sex, age, year of publication, and handedness classification, have a moderating effect in the putative stuttering-handedness relationship?

In terms of question (1), we hypothesize individuals who stutter will have elevated atypical (left, mixed, or non-right)-handedness compared to control participants. In terms of question (2), we refrain from forming concrete hypotheses, as our analysis will be exploratory in nature. Furthermore, this study aims to explore the presence of heterogeneity and small study bias among the datasets.

## Methods

The reporting of the meta-analyses follows the guidelines of the PRISMA statement (Page et al., 2021). Where more appropriate to our field of research, the NIRO-SR (Topor et al., 2020) guidelines were followed (e.g., item A3 for formulating the research question). The PRISMA 2020 Main Checklist as well as the PRISMA 2020 Abstract Checklist are to be found in the Supplementary Material.

### Study Search

The search strategy aimed for completeness. The studies that were included in the meta-analyses were located via a multi-step process:

(i) Via* databases*: The electronic databases PubMed, Scopus, and PsychInfo were searched on June 29th 2021 from inception, in “All Fields”. The search was updated on June 13th 2023 to cover the years 2021–2023. The search terms used were the following:
  *PubMed* (*accessed through *https://pubmed.ncbi.nlm.nih.gov/): ((handedness) OR (handed) OR (hand preference)) OR (hand skill)) AND (stutter*).
  *Scopus* (*accessed through scopus.com*)*:* ((ALL (handedness) OR ALL (hand AND skill) OR ALL (hand AND preference) OR ALL (handed)) AND ALL (stutter*)).
  *PsychInfo* (*accessed through EBSCOhost*): Four searches were conducted: i) (stutter or stuttering) AND Handedness, (ii) (stutter or stuttering) AND handed, (iii) (stutter or stuttering) AND hand skill, (iv) (stutter or stuttering) AND hand preference.

(ii) Via* other methods*: The reference lists of the included articles and of an MSc thesis on handedness in stuttering (Valtou, 2017) were hand-searched. No pertinent review papers we could consult were located. Moreover, e-mail requests for missing data and unpublished datasets were sent to the authors of the included articles (or of the papers for which inclusion was contingent on obtaining data that were not reported in the manuscript). However, in a number of cases, the email addresses could not be retrieved.

### Study Selection

The open-source reference management software Zotero v.5.0.96.3 (Roy Rosenzweig Center for History and New Media, 2021) was used to create a database of all retrieved records and to identify and merge duplicates. Titles, abstracts, and full texts were screened sequentially. AKP and CS performed the study selection independently and any inconsistencies were resolved (i) by taking the records to the next stage of the review in the titles and abstracts screening stages, even if only one reviewer accepted them or was unsure and (ii) through discussion in the full-text stage. No differences remained after discussion, only cases of uncertainty which were resolved by a third reviewer, specializing in meta-analyses on handedness (MPP). Studies that were excluded at the full-text stage together with the reasons for exclusion are listed in Suppl. Table 1.

### Inclusion and Exclusion Criteria

The following criteria were set for inclusion of an individual study in the meta-analyses:

1. *Participants:* To be considered for inclusion, studies had to measure the handedness in individuals who stutter, otherwise healthy (i.e., without comorbidity) as well as in an age-matched control group of individuals who do not stutter.
2. *Sufficient handedness data*: Handedness data had to be presented in a usable way for the analysis or they had to be provided by the authors.
3. *No selection of participants on the basis of handedness*: Studies that either encouraged or discouraged left-handers to participate were excluded. In cases of studies where handedness was reported to be matched between individuals who stutter and controls (e.g., Desai et al., 2017), we contacted the authors to make sure this was not on purpose.
4. *Publication type*: No case studies of individuals who stutter were included. Review studies were also excluded.
5. *Publication language*: Reports had to be written in English, German, or Greek to be included (i.e., the languages spoken by the research team). However, we did not come across any German or Greek reports during study search.

### Data Extraction

AKP and CS performed the study extraction independently and any inconsistencies were resolved through discussion. Any remaining ambiguities were resolved by a third reviewer (MPP). Missing data were not replaced. The following data were extracted from each study:

1. *Handedness data*: The number of left-, mixed-, non-right-, or right-handed participants were extracted for each group (individuals who stutter and controls). The mean handedness scores (with their standard deviations) of individuals who stutter and controls were also extracted when provided.
2. *Year of publication*: The year of publication of the study was extracted and entered into the database numerically. A higher prevalence of left-handedness has been shown in more recent studies compared to very early studies (Papadatou-Pastou et al., 2020). Thus, year of publication was used as a proxy for secular change in the hypothesized stuttering-handedness relationship. Indeed, previous meta-analyses on handedness and its relationships have also used the year of publication as a moderator (e.g., Markou et al., 2017; Ntolka & Papadatou-Pastou, 2018; Packheiser et al., 2021).
3. *Location*: The country where the study was conducted was extracted. The categories used were USA (coded with 1), Europe (2), East Asia (3), rest of Asia (4). The study by Okasha et al. (1974) and the study by Ardila et al. (1994) which were conducted in Egypt and Colombia, respectively, did not fit any of these categories and were not included in the analysis.
4. *Main purpose of the study*: Whether the main purpose of the study was to measure handedness differences between individuals who stutter and controls was extracted using a yes (= 1) / no (= 2) coding.
5. *Sex of the participants*: The sex of the participants in both groups was extracted as male or female (no study reported information on other gender groups). However, only eight studies providing categorical data (Brosch et al., 1999; Cavenagh et al., 2015; Jenson et al., 2018; Max & Yudman, 2003; Mohammadi & Papadatou-Pastou, 2020; Olander et al., 2010; Records et al, 1977; Rogic Vidakovic et al., 2016) and three providing continuous data (Kronfeld-Duenis et al., 2016; Max & Yudman, 2003; Mohammadi & Papadatou-Pastou, 2020) broke down information on handedness and stuttering by sex. Therefore, following previous work (e.g., Packheiser et al., 2021; Papadatou-Pastou et al., 2021) and as per our pre-registration, we also extracted as a proxy the percentage of male participants in each study (sex ratio; first the ratio was calculated for the individuals who stutter and the control participants and then it was averaged between the two groups).
6. *Mean age of the participants*: The mean age of the participants in both groups was extracted and the average between the two groups was entered into the database numerically. When only an age range was reported, then the middle of the range was recorded (e.g., Okasha et al., 1974). In Porfert and Rosenfield (1978), only median age was reported and this was recorded here.
7. *Handedness classification*: Studies either reported continuous handedness scores or classified their participants categorically, using the following classifications: right vs. left (R-L), right vs. non-right (R-nonR), right vs. mixed vs. left (R-M-L), or they used other 3-way classifications that were here coded as R-M-L, namely R-no preference-L, R-ambidextrous-L, R-Latent L-manifest L, and R-Left changed to Right-Left. One study (Mohammadi & Papadatou-Pastou et al., 2020) reported both R-L (used in the left-handedness [forced choice] meta-analysis) and R-M-L classifications (used in the rest of the meta-analyses). Chrysanthis (1947) used a R-L classification. However, he further broke down the left category into (a) left-hand writers, (b) ambidextrous writers, and (c) right-hand writers with characteristics of left-handedness. Here, the (b) and (c) groups were considered mixed-handers, thus for Chrysanthis (1947) we used a R-M-L classification.
8. *Handedness measurement*: Most studies reported using the Edinburgh Handedness Inventory (EHI; Oldfield, 1971) for measuring handedness (coded = 1), but other measures were also used (coded = 2).
9. *Self-report of handedness*: Whether handedness was self-reported or not (i.e., was observed or was assessed by filial report) was extracted using a yes/no coding.
10. *Method of assessing stuttering*: The method via which stuttering was assessed ranged from self-report to speech evaluation. We coded as 1 the studies that used speech evaluation for stuttering assessment and as 2 studies that did not evaluate speech evaluation (e.g., used a questionnaire or stuttering was self-reported). Methods of assessment were so varied that no other meaningful classifications could be used.
11. *Severity of stuttering*: Severity was extracted as reported, using the terms (very) mild, moderate, or (very) severe.

### Pre-registration

The study was pre-registered on PROSPERO before the authors started to identify eligible studies for inclusion (https://shorturl.at/BJSZ2). The time-stamped date of registration in PROSPERO was June 14th, 2021. Pre-registration was conducted in order to ensure unbiased data analysis.

### Deviations from the Pre-registered Protocol

The only deviations from the pre-registered protocol in terms of study identification and data extraction were that (i) the reference list of Valtou’s (2017) MSc thesis was also hand-searched, (ii) AKP and CS did the study search and the data extraction in the place of MMV and MPP, (iii) review studies were excluded, and (iv) the control groups were preregistered to be age-matched, but in the majority of cases, no information on age-matching was provided in the papers or papers reported only one age range for the whole sample (both stutterers and controls). In Records et al. (1977), age is not reported at all.

In terms of data analysis, this followed the pre-registered protocol accurately, but more details (e.g., the type of adjustment used for calculating the overall effect size) and some extra analyses (namely the sensitivity analysis, the use of drapery plots as well as outlier and influential cases identification) are reported in the manuscript. The only substantial difference is that we pre-registered that we would investigate differences in “left-handedness,” but, here, we decided to separately analyze two manifestations of left-handedness: left-handedness extreme (corresponding to left-handers as identified in right-mixed-left categorizations) and left-handedness by forced choice (corresponding to left-handers as identified in right-left categorizations). The former manifestation, left-handedness extreme, is a more conservative and strict classification of left-handedness compared to the latter, left-handedness by forced choice, because in the case of left-handedness by forced choice, participants must choose between being classified as either left- or right-handed, with no middle category available (as is the case in R-M-L categorizations, which is where left-handedness extreme stems from). Consequently, a number of mixed-handed participants could end up being classified as left-handers. In other words, we expect that the degree of left-handedness is higher in the left-handedness extreme manifestation compared to the left-handedness by forced-choice manifestation. We further replaced the pre-registered term “publication bias” with the term “small study bias,” which is more accurate as the (pre-registered) tests used for this analysis look for systematic relationships between a measure of study size (e.g., standard error) and its effect. Lastly, we used the term “stuttering assessment” in the place of “stuttering diagnosis,” as it describes the participant selection procedure more accurately.

### Statistical Analysis

The statistical analysis was carried out using R (v. 4.2.1 for macOS) and RStudio (2022.07.1 Build 554; R Core Team, 2022; RStudio Team, 2022) using the “meta” (v. 5.5–0; Balduzzi et al., 2019), “metafor” (v. 3.4–0; Viechtbauer, 2010), “dmetar” (v.0.0.9000; Harrer et al., 2019), and “tidyverse” (v. 1.3.2; Wickham et al., 2019) packages.

Analysis was performed by MPP and assessed for reliability by SO. We have two main outcomes:

(i) The odds ratio (OR) of atypical handedness prevalence between the two groups (individuals who stutter vs. controls) with corresponding 95% confidence intervals (CI), in the case of studies that provided categorical handedness data. An odds ratio value of 1.0 corresponds to the null hypothesis of no handedness differences between the two groups, whereas values greater than 1.0 indicate a larger proportion of atypical handedness among individuals who stutter. The OR can also be transformed to a simple proportion, using the formula IS = IWDNS x OR/ [1 + IWDNS(OR-1)], so that the findings can be more intuitively grasped (IS = individuals who stutter, IWDNS = individuals who do not stutter). Following previous work (e.g., Markou et al., 2017; Nastou et al., 2022), we provide separate analyses for different handedness classifications. These handedness categories were defined as follows:
  *Left-handedness* (*forced choice*): Participants who were classified as left-handed in R-L classifications were included in this analysis (and compared to the total number of participants).
  *Left-handedness* (*extreme*): Participants who were classified as left-handed in R-M-L classifications were included in this analysis (and compared to the total number of participants).
  *Mixed-handedness*: Participants who were classified as mixed-handed were included in this analysis (and compared to the total number of participants).
  *Non-right-handedness*: Participants who were classified as left- or mixed-handed in either R-L or R-M-L classifications were included in this analysis, as well participants classified as non-right in R-nonR classifications (and compared to the total number of participants). Thus, this was the most inclusive meta-analysis.

(ii) The standardized difference in mean handedness scores (Cohen’s *d*) between individuals who stutter and controls with corresponding 95% CI, in the case of studies that provided continuous handedness data. A *d* value of zero corresponds to the null hypothesis of no handedness differences between the two groups, whereas values greater than zero indicate a larger proportion of atypical handedness among individuals who stutter. Standardized mean differences are often interpreted using the conventions by Cohen (1988), whereby effect sizes equaling 0.20, 0.50, and 0.80 are considered low, moderate, and large, respectively. Of note, following previous meta-analysis on handedness (e.g., Papadatou-Pastou and Tomprou, 2015; Markou et al., 2017; Nastou et al., 2022), we decided not to convert the ORs calculated for categorical handedness data to* d.*

For both outcomes (OR and *d*) the effect sizes with their corresponding two-tailed 95% CI were calculated for each dataset independently. They were then combined using a random effects model to provide a pooled effect size and a test (*t*) for the overall effect (with its corresponding *p* value). A random effects model rather than a fixed effects model was put forward given the variability in handedness measures used as well as in the ways that stuttering was assessed. The Hartung-Knapp adjustment (Knapp & Hartung, 2003) was applied to calculate the CI around the pooled effect, as it has been suggested to reduce the chance of false positives, especially when the number of studies is small (IntHout et al., 2014; Langan et al., 2019). The Mantel–Haenszel method (Mantel & Haenszel, 1959; Robins et al., 1986) was used to calculate the weights of studies in the categorical data meta-analyses without continuity correction, as suggested by Higgins et al. (2019). The inverse variance method was used in the continuous data meta-analysis.

Moreover, we explored the presence of heterogeneity using the *Q* statistic, the *I*^2^ index (with 95% CI), and the τ^2^ statistic. The *Q* statistic is used to ascertain whether the primary level effect sizes estimate a common population effect size and the *I*^2^ index is interpreted as the percentage of total variation across studies that is due to heterogeneity rather than chance. Higgins et al. (2003) have proposed that levels of 25%, 50%, and 75% may be described as low, moderate, and high, respectively. The τ^2^ statistic represents the variance of the distribution of the true effect sizes and is thus an estimate of the between-study variance. Tau-squared was estimated using the Paule-Mandel method (Paule & Mandel, 1982) for categorical data and the restricted maximum likelihood estimator (Viechtbauer, 2005) for continuous data, as recommended by Veroniki et al. (2016). Prediction intervals, the range into which we can expect the effects of future studies to fall based on present evidence, are further reported.

When heterogeneity was found to be present, we conducted moderating variables analysis, including the following pre-registered variables: year of publication, location, sex ratio, mean age, handedness classification, handedness measure, stuttering assessment, main purpose of the study, and whether handedness was self-reported or not. In order to perform any moderating variables analysis, at least 5 data points per level of the moderator (or at least 5 studies in the case of continuous variables) were needed, as pre-registered. Severity of stuttering could not be used as a moderator, although pre-registered, as only three studies (Maruthy et al., 2017; Olander et al., 2010; Rogić Vidaković, 2016) broke down their handedness data according to the severity of stuttering.

In terms of sensitivity analysis, if one study had a weight in the analysis of 25% or above, the meta-analysis was repeated without this study to evaluate the stability of the population-effect size, following Westerhausen and Papadatou-Pastou (2022). We also performed outlier identification, whereby a study was identified as an outlier if its 95% CI did not overlap with the 95% CI of the pooled effect. Influential cases identification was further conducted through producing a Baujat plot, an overall influence diagnostic plot, and two leave-one-out meta-analysis plots (one sorted by effect size and the other by *I*^2^) for each meta-analysis (see Harrer et al., 2021, for more details). For the continuous data meta-analysis, the overall effect was recalculated using the Paule-Mandel method as the tau-squared estimator, before the influential cases identification analysis was performed.

Forest plots were used to depict all the information visually together with drapery plots (Rücker & Schwarzer, 2021). While forest plots display CI using a fixed significance threshold (*p* < 0.05 in our case), drapery plots are based on *p* value functions. Thus, they plot a continuous curve which shows the CI for varying values of *p*.

A detailed risk-of-bias analysis (a term also to be found as critical appraisal, certainty assessment, or quality assessment) was not deemed necessary in the context of these meta-analyses. Risk-of-bias analysis is suited to meta-analyses of studies assessing an intervention (therefore the presence of elements like the blinding of participants and randomization need to be assessed) or an experimental manipulation (therefore elements like blinding of the experimenters need to be assessed), thus not suited to our research question. However, we did include the following elements to our methodology to ensure the quality of our findings:

(i) We tested for the presence of small study bias using Egger’s *t* test, the Funnel plot, and the Trim-and-Fill method (Duval & Tweedie, 2000).
(ii) In terms of quality of individual studies, we only included published studies that may be assumed to have sufficient quality as a result of peer-review processes (although we did ask authors to provide extra data not reported in the published paper in some cases, as shown in Table 1). Moreover, only studies including a control group were eligible for inclusion. This ensured that handedness was assessed in both individuals who stutter and controls using the same handedness measure. Therefore, the effect size was independent of the base rate of handedness in each study.
(iii) We checked for various methodological qualities of our included studies (e.g., handedness measurement used) in the context of moderator analyses.

**Table 1:** Overview of studies included in the meta-analyses [*EHI* Edinburgh Handedness Inventory (Oldfield, 1971), *LHfc* left-handedness (forced choice), *LHe* left-handedness (extreme), *MH* mixed-handedness, *NonRH* non-right-handedness, *Cont* continuous data]

**Study**	**Handedness classification**	**Stuttering assessment**	**Handedness measurement**	**Males %**	**Mean age in years**	**Included in meta-analysis**					**Notes**
						**LHfc**	**LHe**	**MH**	**NonRH**	**Cont**	
Ardila et al., 1994	R-M-L	Adapted version of the questionnaire of Roberts et al. (1990)	Self report (“Are you right-handed, left-handed, or ambidextrous?”)	52.16	22.55		●	●	●		
Arnold et al., 2011	R-L	Speech evaluation & Stuttering Severity Instrument 3 (Riley, 1994)	EHI	66.67	4.54	●			●		
Arnstein et al., 2011	R-M-L	Stuttering Severity Instrument 3 (Riley, 1994)	Self-report on a three-choice item (“right-handed, “left-handed”, “mixed”)	96.43	30.65		●	●	●		
Brosch et al., 1999	R-No preference-L	Stuttering Severity Instrument (Riley, 1972)	Laterality tests	58.05	5.25		●	●	●		Children were classified as either right- or left-handed on the basis of how they threw a ball, drew a picture, grasped an object and kicked a ball. If two of the four tests were done with different hands or if they changed hands when the tests were repeated, the children were classified as ‘no hand preference’
Bryngelson & Rutherford, 1937	R-Ambidextrous-L	Speech evaluation	Not reported	44.59	10		●	●	●		
Bryngelson, 1940	R-Ambidextrous-L	Speech evaluation	Not reported	37.18	24.5		●	●	●		The sum of the percentages reported in Table 1 for the individuals who stutter was 104%, so we extrapolated to 100%
Cavenagh et al., 2015	R-No preference-L	Speech evaluation & parental report & Stuttering Severity Instrument (Riley, 1994)	Parental report (“Is your child left- or right-handed?”)	60.08	4.04		●	●	●		
Chang et al., 2015	R-Ambidextrous-L	Speech evaluation & Stuttering Severity Instrument 4 (Riley, 2009)	EHI	55.98	6.35		●	●	●	●	Categorical data are reported for 47 individuals who stutter and 42 controls, but continuous data are reported for 37 individuals who stutter and 40 controls
Choo et al., 2016	R-Ambidextrous-L	Stuttering Severity Instrument 4 (Riley, 2009)	EHI	58.05	5.77		●	●	●		The handedness of two individuals who stutter and one control could not be determined. Authors kindly provided more data
Chrysanthis, 1947	R-M-L	Speech evaluation	Not reported	Not reported	10.5		●	●	●		Right-handed writers—Right-handed writers with characteristics of left-handedness—ambidextrous writers—Left-handed writers. The two middle categories were considered "mixed-handed" for the purposes of these analyses
Coalson & Byrd, 2015 (dataset 1)	Continuous	Self report & speech evaluation (O’Brian et al., 2004)	EHI	63.63	21					●	Authors kindly provided the data
Coalson & Byrd, 2015 (dataset 2)	Continuous	Self report & speech evaluation (O’Brian et al., 2004)	EHI	63.63	22.5					●	Authors kindly provided the data
Connally et al., 2014	R-L	Stuttering Severity Instrument 3 (Riley, 1994)	Not reported	67.29	23.45	●			●		
Corbera et al., 2005	R-L	DSM-IV (American Psychiatric Association (APA), 1994) & Self-report questionnaire [Conduct and Attitude Scale for the Assessment of Disfluencies (CASAD)]	EHI	83.97	22.75	●			●		
Cross, 1987	R-L	Profile of Stuttering Behavior (Van Ripen, 1971) (not clear if questionnaire was used or based on sample evaluation)	EHI	100	25.5	●			●		
Cykowski et al., 2010	Continuous	Self report & speech evaluation (Ingham et al., 1999)	EHI	100	30.7					●	
Dellatolas et al., 1990 (dataset 1)	Continuous/R-L	Self report	10 questions regarding hand preference	51.75	34.5					●	The LIs are reported in the paper in a way that higher LIs corresponded to stronger left-handedness. We have transformed the scores so that higher scores correspond to stronger right-handedness, as was the case in the rest of the studies
Dellatolas et al., 1990 (dataset 2)	Continuous/R-L	Self report	10 questions regarding hand preference	100	20.5					●	As above
Desai et al., 2017	R-Non-right	Speech evaluation & Assessment of the Child’s Experience of Stuttering (ACES) (Yaruss et al., 2006) & Overall Assessment of the Speaker’s Experience of Stuttering (OASES; Yaruss & Quesal, 2006)	EHI	56.62	19.49				●		
Frankford et al., 2021	R-Ambidextrous-L	Self report & Stuttering Severity Instrument (Riley, 2009)	EHI	66.67	29.28		●	●	●		
Friedmann, 1937	R-Latent L-Manifest L	Not reported	Not reported	Not reported	Not reported		●	●	●		The sum of percentages for the individuals who stutter was 102.8%, so we extrapolated to 100%. Both children and adults who stutter were included, whereas the control group consisted of children, thus the groups are not age-matched
Gough et al., 2018	R-L	Stuttering Severity Instrument (Riley, 1994, 2009)	Not reported	77.60	25.58	●			●		
Hampton & Weber-Fox, 2008	R-L	Stuttering Severity Instrument 3 (Riley, 1994)	EHI	72.73	34.68	●			●		
Hand & Haynes, 1983	R-Sinistral (L)	Self report & Stuttering Severity Instrument (Riley, 1972)	Neurosensory Center Handedness Inventory (Varney & Benton, 1975)	100	25	●			●		
Jansson-Verkasalo et al., 2014	R-L	Speech evaluation & Stuttering Severity Instrument 3 (Riley, 1994)	Not reported	79.17	7.54	●			●		
Jenson et al., 2018	R-L	Stuttering Severity Instrument 4 (Riley, 2009)	EHI	70.83	25.21	●			●		
Kaganovich et al., 2010	R-L	All criteria by Yairi & Ambrose, 1999 met	Parental report & 5 questions from EHI	69.44	4.83	●			●		
Koenraads et al., 2020	R-L	Parental report	ΕΗΙ	64.08	10	●			●		Data from recovered stutterers are also reported, but we only included data from individuals with persistent stuttering
Kronfeld-Duenias et al., 2016	R-L	Self report & Speech evaluation & Stuttering Severity Instrument 3 (Riley, 1994)	EHI	82.11	32.5	●			●	●	Authors kindly provided raw data on EHI. We classified it into R-L using L > = 50 and R > 50
Lescht et al., 2022	R-Ambidextrous-L	Parental report & Stuttering Severuty Instrument (Riley, 2009)	Abbreviated EHI	57.89	5.77		●	●	●		
Liman et al., 2021	Continuous	Stuttering Severity Instrument 3 (Riley, 1994)	EHI	70	26.81					●	
Maruthy et al., 2017	R-L	Self report & Stuttering Severity Instrument 3 (Riley, 1994)	Self report	90	21.1	●			●		
Max & Yudman, 2003	R-M-L	Self report & Stuttering Severity Instrument 3 (Riley, 1994)	Lateral Preference Inventory (LPI; Coren, 1993) & Self report	70	34.1		●	●	●	●	We classified participants using information from Table 1 by assigning right for > 3 “right” responses, left for > 3 “left” responses and assigning the rest as mixed. We calculated the continuous score again using information from Table 1 and assigning 0 to “left”, 1 to” either” and 2 to “right” responses
Maxfield et al., 2015	R-L	Self report & speech evaluation & Overall Assessment of the Speaker's Experience with Stuttering (OASES) (Yaruss & Quesal, 2006)	Not reported	71.05	25.46	●			●		
Mohammadi & Papadatou-Pastou, 2020	R-L & R-M-L	Speech evaluation & Stuttering Severity Instrument 3 (Riley, 1994)	EHI	72.9	6.55	●	●	●	●	●	Participants were classified as R-M-L using two cut-offs (- ± 80 and ± 60). Here we report the ± 80 cutoff point The R-L classification was used in the LHfc meta-analysis and the R-M-L classification in the rest of the categorical meta-analyses
Murase et al., 2016	R-L	Self report & Speech evaluation	H.N. Handedness Inventory (Hatta, 1996)	80.77	32.65	●			●		
Okasha et al., 1974	R-Left changed to R- L	Speech evaluation	Not reported	Not reported	9		●	●	●		The "middle" category consists of "those who started left-handed and changed to right-handedness" as reported in the manuscript
Olander et al., 2010	R-L	Speech evaluation (Yairi & Ambrose, 1999)	5 questions from EHI	72.85	4.88	●			●		
Piispala et al., 2017	R-L	Not reported	Not reported	81.58	7.8	●			●		
Porfert & Rosenfield, 1978	R-Non-right	Self report	Self report (“Are you right-, mixed-, or left-handed?”)	63.85	20.8				●		Handedness information on past stutterers and questionable stutterers were not included in the meta-analysis
Preibisch et al., 2003a	R-Non-right	Recruitment from a treatment program	EHI	75.63	31				●		
Preibisch et al., 2003b	R-Non-right	Self report & Speech evaluation	EHI	100	31				●		
Records et al., 1977	R-Ambidextrous-L	Not reported	Handedness questionnaire	55.45	Not reported		●	●	●		Data were extracted from Table 3 (there were slight inconsistencies with other parts of the manuscript)
Rogic Vidakovic et al., 2016	R-L	Recruitment from clinics & Stuttering Severity Instrument 3 (Riley, 1994)	EHI	70.74	27.56	●			●		
Sommer et al., 2002	R-Non-right	Confirmation of diagnosis by pathologists/neurologists	EHI	70	30.3				●		
Toyomura et al., 2011	R-L	Stuttering Severity Instrument 4 (Riley, 2009)	Not reported	91.67	27.5	●			●		
Toyomura et al., 2018	R-Ambidextrous-L	Recruitment from self-help group & Stuttering Severity Instrument 4 (Riley, 2009)	EHI	75	29.7			●	●		
Toyomura et al., 2020	R-L	Speech evaluation	EHI	65.63	23.75	●			●		
Vanhoutte et al., 2015	R-L	Self report & Speech evaluation & Stuttering Severity Instrument 4 (Riley, 2009)	EHI	72.29	29.4	●			●		
Vreeswijk et al., 2019	Continuous	Recruitment from stuttering therapy and support groups & Speech evaluation & Stuttering Severity Instrument (Sandrieser & Schneider, 2008)	EHI	79.29	29.29					●	
Walsh et al., 2019	R-L	Speech evaluation (Yairi & Ambrose, 1999) & Test of Childhood Stuttering (TOCS; Gillam et al., 2009)	EHI	76.43	4.65	●			●		Authors kindly provided handedness data
Watkins et al., 2008	R-L	Stuttering Severity Instrument 3 (Riley, 1994)	Not reported	63.33	18	●			●		
Whillier et al., 2018	Continuous	Stuttering Severity Instrument 3 (Riley, 1994)	EHI	70.92	25.09					●	
Wieland et al., 2015	Continuous	Speech evaluation & Stuttering Severity Instrument 4 (Riley, 2009)	EHI	47.06	8.75					●	

## Results

**Fig. 1:**
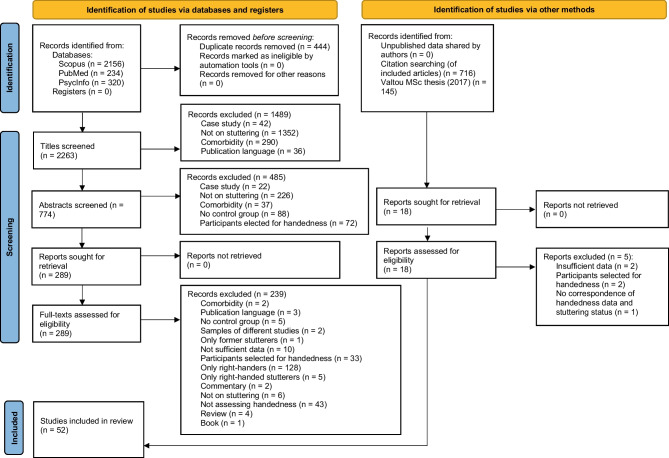
Flow diagram of the search and selection process, adapted from the PRISMA flow diagram (Page et al., 2021; editable file downloaded from http://prisma-statement.org/PRISMAStatement/FlowDiagram). Note: Some records were excluded for more than one reason

Figure 1 is a flow diagram of the search and selection process. Data from *k* = 54 datasets (from *k* = 52 studies) published between 1937 and 2022 were included in one or several meta-analyses, as indicated in Table 1. The number of records included in the categorical data meta-analyses was *k* = 45 datasets (from *k* = 45 studies) and the number of records included in the continuous data meta-analyses was *k* = 13 datasets (from *k* = 11 studies). Chang et al. (2015), Kronfeld-Duenias et al. (2016), Max and Yudman (2003), and Mohammadi and Papadatou-Pastou (2020) contributed to both the categorical and the continuous data meta-analyses. One paper was authored by a member of our team [MPP]. In total, *n* = 19,738 participants (*n* = 2590 individuals who stutter, *n* = 17,148 control individuals) were included in the meta-analyses.

### Categorical Data Meta-Analyses

#### Left-Handedness (Forced Choice)

**Fig. 2:**
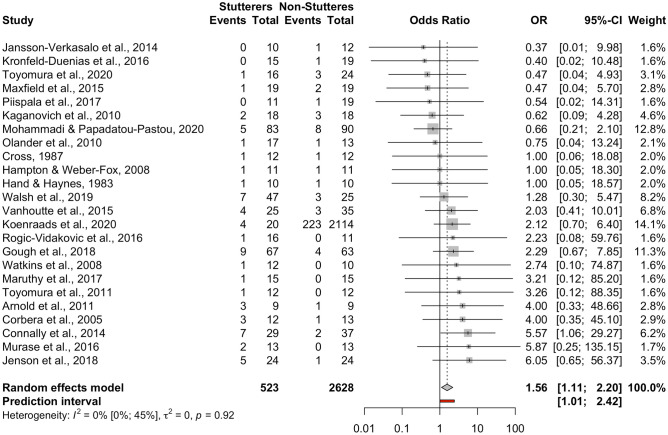
Forest plot of the individuals-who-stutter to individuals-who-do-not-stutter odds ratios for the left-handedness (forced choice) comparison. In the plot, the 95% confidence interval for each study is represented by a horizontal line and the point estimate is represented by a square. The confidence intervals for totals are represented by a diamond shape at the bottom of the plot

This meta-analysis included *k* = 24 studies adding up to *n* = 3151 participants (*n* = 523 individuals who stutter, *n* = 2628 control individuals). Only one study (Connally et al., 2014) appeared to have a significant effect size (*p* < 0.05). The pooled OR was OR = 1.56 [95% CI: 1.11; 2.20], *t* = 2.69, *p* = 0.01 (see forest plot, Fig. 2, and drapery plot, Suppl. Fig. 1). This suggests that there is evidence that individuals who stutter have higher prevalence of left-handedness (forced choice) compared to controls. There was no heterogeneity among the datasets, *Q*(23) = 14.10, *p* = 0.92, with no inconsistency between studies, *I*^*2*^ = 0.00% [95% CI: 0.00%; 44.60%]. The between-study heterogeneity variance was τ^2^ = 0 [95% CI: 0.00; 0.19], suggesting a 95% prediction interval from 1.01 to 2.42 around the mean effect. The fact that the lower bound of the prediction interval is close to the odds ratio value of 1.0 calls for some caution when interpreting the findings of this meta-analysis. When comparing males and females in the four studies that broke down their data by sex (Jenson et al., 2018; Mohammadi & Papadatou-Pastou, 2020; Olander et al., 2010; Rogic Vidakovic et al., 2016), no sex differences were found, *Q*(1) = 0.35, *p* = 0.55.

#### Small Study Bias

Neither the inspection of the funnel plot (Suppl. Fig. 2) nor Egger’s test (intercept = −0.12 [95% CI:−0.96; 0.72], *t* = -0.29, *p* = 0.77) suggested a small study bias. One study was added using the Trim-and-Fill method to make the funnel plot symmetrical, making the evidence of a difference in ORs stronger (*OR* = 1.60 [95% CI: 1.14; 2.25], *t* = 2.84, *p* = 0.009), although the prediction interval (95% PI: 1.04, 2.47) is again very close to one.

#### Sensitivity Analysis

No outliers were detected. Mohammadi and Papadatou-Pastou (2020) was identified as influential in the Baujat plot (Suppl. Fig. 3), with some evidence also shown in the two leave-one-out meta-analysis plots (Suppl. Figs. 4 and 5). The influence diagnostics plot (Suppl. Fig. 6) also identified the study of Kaganovich et al. (2010) as well as Mohammadi and Papadatou-Pastou (2020) as influential cases. Without these studies, strong evidence of a difference in ORs was found (*OR* = 1.88 [95% CI: 1.34; 2.65], *t* = 3.85, *p* < 0.001), as in the original analysis. The 95% CI of the prediction interval was 1.16 to 3.06, thus not including one. Overall, the evidence is clear that differences exist in the prevalence of left-handedness (forced choice) between individuals who stutter and controls. Table 2 summarizes the results of the sensitivity analysis for all meta-analyses.

**Table 2:** Sensitivity analysis (*PI* prediction interval, *CI* confidence interval)

	**Categorical data analysis**						
	**N datasets**	**N stutterers/non-stutterers**	**Median *N* stutterers/non-stutterers**	**OR [95% CI]**	***p***	**95% PI**	***I***^**2**^** [95% CI]**
Left-handedness (forced choice)	24	523/2628	15.5/16.5	1.56 [1.11; 2.20]	0.01*	1.01; 2.42	0.00% [0.00%; 44.60%]
Kaganovich et al. (2010)* & *Mohammadi and Papadatou-Pastou (2020) removed	*22*	*422*/*2520*	*15/ 14*	*1.88 [1.34; 2.65]*	< 0.001*	*1.16; 3.06*	*0.00% [0.00%; 46.20%]*
Left-handedness (extreme)	16	1181/3938	57/64	1.24 [0.65; 2.40]	0.49	0.15; 10.03	66.70% [43.60%; 80.30%]
Chrysanthis (1947)* removed*	*15*	*1160*/*2826*	*66*/*53*	*1.04 [0.78; 1.40]*	*0.77*	*0.76; 1.43*	*0.00% [0.00%; 53.60%]*
Mixed handedness	16	1189/3942	57/64	1.12 [0.54; 2.34]	0.74	0.12; 10.98	63.30% [37.20%; 78.60%]
*Bryngelson (*1940*) removed*	*15*	*1111*/*3864*	*47*/*53*	*0.96 [0.53; 1.77]*	*0.90*	*0.19; 4.89*	*56.60% [22.70%; 75.60%]*
*Bryngelson (*1940*) & *Bryngelson and Rutherford (1937)* removed*	*14*	1037/3790	*45*/*48*	*0.70 [0.45; 1.10]*	*0.11*	*0.33; 1.50*	*12.20% [0.00%; 50.70%]*
Non-right-handedness	45	1774/8418	19/19	1.42 [1.11; 1.81]	0.007**	0.71; 2.80	28.50% [0.00%; 50.70%]
*Bryngelson (*1940*) & *Chrysanthis (1947)* removed*	*43*	*1675/7228*	*18/19*	*1.12 [0.92; 1.35]*	*0.23*	*0.92; 1.36*	*0.00% [0.0%0; 35.10%]*
	**Continuous data analysis**						
				***d [*95% CI]**	***p***	**95% PI**	***I***^***2***^** [95% CI]**
Main Analysis	13	961/8889	14/17	−0.06 [− 0.18; 0.05]	0.26	−0.30; 0.17	0.00% [0.00%; 56.60%]
Dellatolas et al. (1990)* study 2 removed*	*12*	*269*/*922*	*15/15*	−*0.004 [*− *0.17; 0.16]*	*0.95*	−*0.27; 0.26*	*0.00% [0.00%; 58.30%]*

*Results significant for a = 0.05

**Results significant for a = 0.01

#### Moderating Variables Analysis

No moderating variables analysis was conducted, due to lack of heterogeneity.

### Left-Handedness (Extreme)

**Fig. 3:**
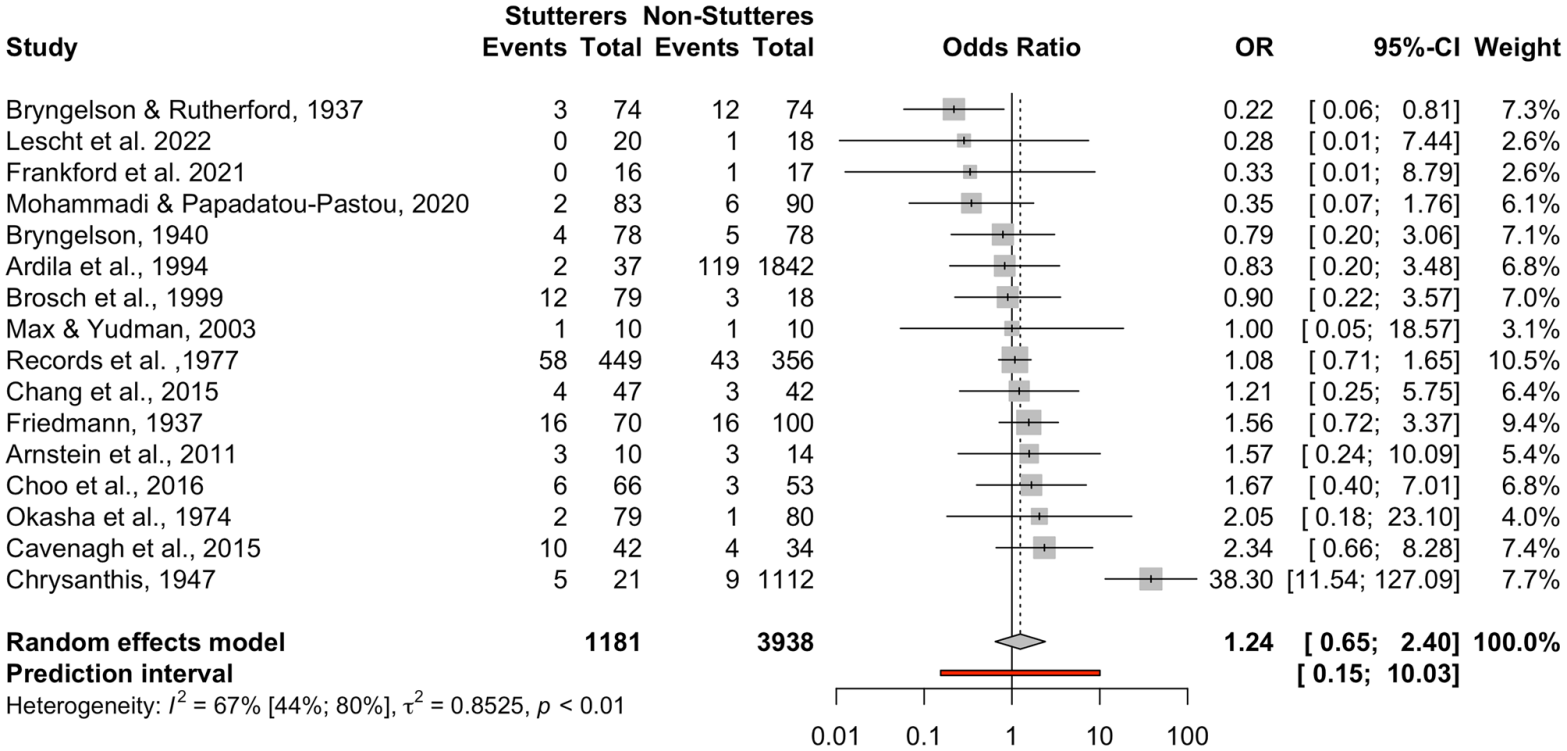
Forest plot of the individuals-who-stutter to individuals-who-do-not-stutter odds ratios for the left-handedness (extreme) comparison

This meta-analysis included *k* = 17 studies adding up to *n* = 5155 participants (*n* = 1199 individuals who stutter, *n* = 3956 control individuals). However, one study (Toyomura et al., 2018) contributed zero left-handers for both groups, thus an odds ratio could not be calculated. After the exclusion of this study, the meta-analysis included *k* = 16 studies adding up to *n* = 5119 participants (*n* = 1181 individuals who stutter, *n* = 3938 control individuals). Only two datasets (Bryngelson & Rutherford, 1937; Chrysanthis, 1947) appeared to have a significant effect size (*p* < 0.05). The pooled OR was OR = 1.24 [95% CI: 0.65; 2.40], *t* = 0.71, *p* = 0.49 (see forest plot, Fig. 3, and drapery plot, Suppl. Fig. 7). Therefore, there is no evidence that individuals who stutter have higher prevalence of left-handedness (extreme) compared to controls. There was evidence of heterogeneity among the datasets, *Q*(15) = 44.99, *p* < 0.001, with high inconsistency between studies, *I*^*2*^ = 66.7% [95% CI: 43.60%; 80.30%]. The between-study heterogeneity variance was τ^2^ = 0.85 [95% CI: 0.26; 2.76], suggesting a 95% prediction interval from 0.15 to 10.03 around the mean effect. When comparing males and females in the five studies that broke down their data by sex (Brosch et al., 1999; Cavenagh et al., 2015; Max & Yudman, 2003; Mohammadi & Papadatou-Pastou, 2020; Records et al., 1977), no sex differences were found, *Q*(1) = 1.83, *p* = 0.18.

#### Small Study Bias

Neither the inspection of the funnel plot (Suppl. Fig. 8) nor Egger’s test (intercept = −0.13 [95% CI:−1.76; 1.51], *t* = −0.15, *p* = 0.88) suggested a small study bias. Four studies were added using the Trim-and-Fill method to make the funnel plot symmetrical, again providing no evidence of a difference in ORs (OR = 1.65 [95% CI: 0.89; 3.07], *t* = 1.71, *p* = 0.10, 95% CI: 0.19; 14.48).

#### Sensitivity Analysis

Chrysanthis (1947) was identified both as an outlier (of note: the OR for this study was 38.30, [95% CI: 11.54; 127.09], when the pooled OR was 1.24, [95% CI: 0.65; 2.40]) and an influential study (see the Baujat plot [Suppl. Fig. 9], the influence diagnostics plot [see Suppl. Fig. 10], and the two leave-one-out meta-analysis plots [Suppl. Figs. 11 and 12]). Without this study, the pooled OR was OR = 1.04 [95% CI: 0.78; 1.40], *t* = 0.30, *p* = 0.77, thus, again, no evidence that individuals who stutter have higher prevalence of left-handedness (extreme) compared to controls was found. Of note, the heterogeneity was now depleted; no heterogeneity was found among the datasets, *Q*(14) = 12.19, *p* = 0.59, with no inconsistency between studies, *I*^*2*^ = 0.00% [95% CI: 0.00%; 53.60%]. The between-study heterogeneity variance was τ^2^ = 0 [95% CI: 0.00; 0.77], suggesting a 95% prediction interval from 0.76 to 1.43 around the mean effect. Overall, the evidence is robust that no differences exist in the prevalence of left-handedness (extreme) between the two groups.

#### Moderating Variables

Although we preregistered that should heterogeneity be found we would search for moderating variables, it was clear from the sensitivity analysis that all the heterogeneity was due to the Chrysanthis (1947) study, a clear outlier. We therefore did not proceed to a moderating variables analysis.

### Mixed-Handedness

**Fig. 4:**
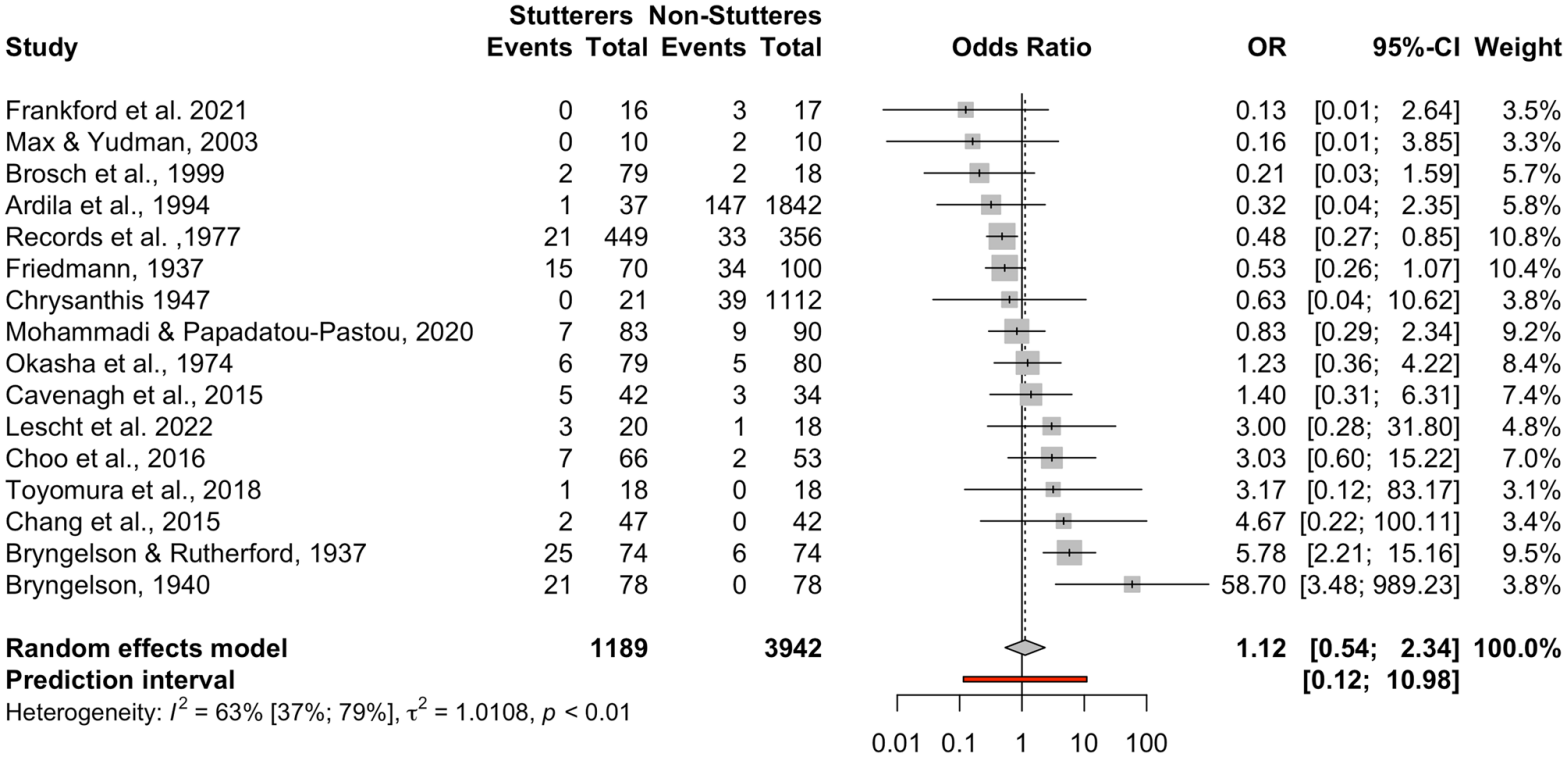
Forest plot of the individuals-who-stutter to individuals-who-do-not-stutter odds ratios for the mixed-handedness comparison

This meta-analysis included *k* = 17 studies adding up to *n* = 5155 participants (*n* = 1199 individuals who stutter, *n* = 3956 control individuals). However, one study (Arnstein et al., 2011) contributed zero mixed-handers for both groups (handedness was assessed by self-report on a three-choice item [“right-handed,” “left-handed,” “mixed”]), thus an odds ratio could not be calculated. After the exclusion of this study, the meta-analysis included *k* = 16 studies adding up to *n* = 5131 participants (*n* = 1189 individuals who stutter, *n* = 3942 control individuals). Only three studies (Bryngelson & Rutherford, 1937; Bryngelson, 1940; Records et al., 1977) appeared to have a significant effect size (*p* < 0.05). The pooled OR was OR = 1.12 [95% CI: 0.54; 2.34], *t* = 0.34, *p* = 0.74 (see forest plot, Fig. 4, and drapery plot, Suppl. Fig. 13). Therefore, there is no evidence that individuals who stutter have higher prevalence of mixed-handedness compared to controls. Heterogeneity was present among the datasets, *Q*(15) = 40.87, *p* < 0.001, with medium to high inconsistency between studies, *I*^*2*^ = 63.30% [95% CI: 37.20%; 78.60%]. The between-study heterogeneity variance was τ^2^ = 1.01 [95% CI: 0.19; 4.35], suggesting a 95% prediction interval from 0.12 to 10.98 around the mean effect. When comparing males and females in the five studies that broke down their data by sex (Brosch et al., 1999; Cavenagh et al., 2015; Max & Yudman, 2003; Mohammadi & Papadatou-Pastou, 2020; Records et al., 1977), no sex differences were found, *Q*(1) = 1.19, *p* = 0.28.

#### Small Study Bias

Neither the inspection of the funnel plot (Suppl. Fig. 14) nor Egger’s test (intercept = 10.83 [95% CI:−0.69; 2.34], *t* = 1.07, *p* = 0.30) suggested a small study bias. One study was added using the Trim-and-Fill method to make the funnel plot symmetrical, but again, no evidence of a difference in ORs was found (OR = 0.94 [95% CI: 0.39; 2.25], *t* = −0.16, *p* = 0.88).

#### Sensitivity Analysis

Bryngelson (1940) was identified as an outlier. Similarly, without this study, no evidence of a difference in ORs was found (OR = 0.96 [95% CI: 0.53; 1.77], *t* = −0.13, *p* = 0.90), as in the original analysis. In terms of influential studies, Bryngelson (1940) and Bryngelson and Rutherford (1937) appear influential in the Baujat plot (Suppl. Fig. 15) as well as in the two leave-one-out meta-analysis plots (Suppl. Figs. 16 and 17), although no studies appear to be influential in the influence diagnostics plot (Suppl. Fig. 18). We therefore repeated the meta-analysis by removing these two studies. Now, the pooled standardized difference in means was OR = 0.70 [95% CI: 0.45; 1.10], *t* = −1.69, *p* = 0.11, again indicating no evidence of a difference in mixed-handedness between individuals who stutter and control individuals. Overall, the evidence is robust that no differences exist in the prevalence of mixed-handedness between the two groups.

#### Moderating Variables Analysis

The results of the moderating variables analysis are shown in Table 3. There were not at least two levels with at least *k* = 5 studies for the self-report, stuttering assessment, and the location variables, therefore these analyses were not conducted. No moderators were identified.

**Table 3:** Moderator variables analysis results for the mixed-handedness meta-analysis

**Variable**	**Levels**	***n*data sets**	**OR**	**95% CI**	**Statistics**
Mean age	Continuous variable	12	n/a	n/a	*F*(1,12) = 0.32, *p* = 0.58
Year of publication	Continuous variable	14	n/a	n/a	*F*(1,14) = 0.61, *p* = 0.45
Sex ratio	Continuous variable	11	n/a	n/a	*F*(1,11) = 4.28, *p* = 0.06
Handedness measurement	EHI	6	1.37	0.5; 4.00	*Q*(1) = 0.26, *p* = 0.61
	Other	10	0.99	0.32; 2.98	
Main purpose	Yes	7	1.46	0.35; 6.04	*Q*(1) = 0.40, *p* = 0.53
	No	9	0.92	0.34; 2.53	

### Non-Right-Handedness

**Fig. 5:**
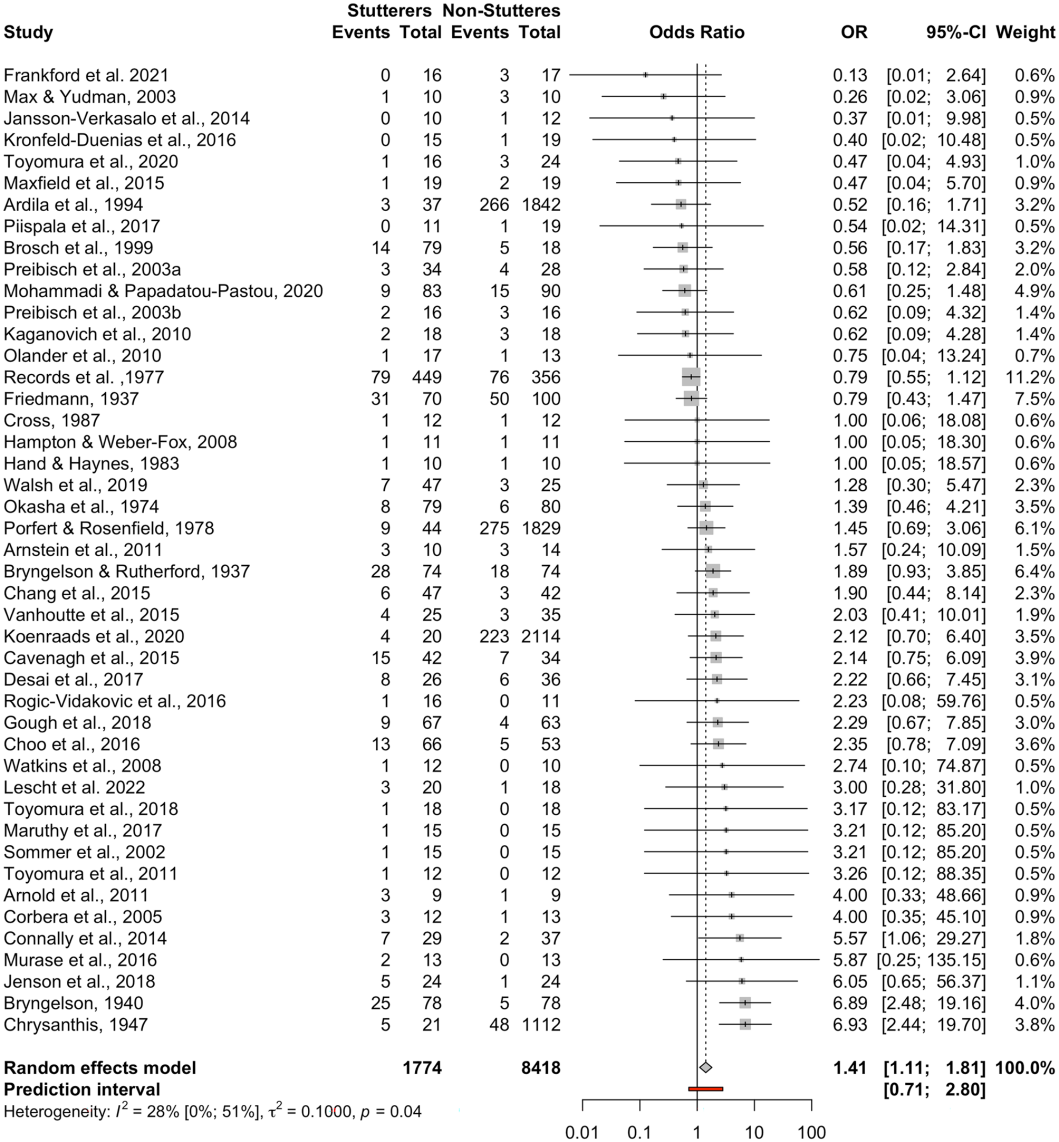
Forest plot of the individuals-who-stutter to individuals-who-do-not-stutter odds ratios for the non-right-handedness comparison

This meta-analysis included all *k* = 45 studies that reported categorical data, adding up to *n* = 10,192 participants (*n* = 1774 individuals who stutter, *n* = 8418 control individuals). Mohammadi and Papadatou-Pastou (2020) classified their participants using both the R-L and the R-M-L classifications. For the purposes of this meta-analysis, the latter classification was used, as grouping left- and mixed-handers together is closer to the notion of non-right-handedness compared to left-handers by forced choice. Only three studies (Bryngelson, 1940; Chrysanthis, 1947; Conally et al., 2014) appeared to have a significant effect size (*p* < 0.05). The pooled OR was OR = 1.42 [95% CI: 1.11; 1.81], *t* = 2.85, *p* = 0.007 (see forest plot, Fig. 5, and drapery plot, Suppl. Fig. 19). Therefore, there is evidence that individuals who stutter have a higher prevalence of non-right-handedness compared to controls. Heterogeneity was marginally present among the datasets, *Q*(44) = 61.53, *p* = 0.041, with low inconsistency between studies, *I*^2^ = 28.50% [95% CI: 0.00%; 50.70%]. The between-study heterogeneity variance was τ^2^ = 0.10 [95% CI: 0.00; 0.43], suggesting a 95% prediction interval from 0.71 to 2.80 around the mean effect. The fact that the prediction interval includes the odds ratio value of 1.0, does not allow for a degree of certainty about the results of this analysis. When comparing males and females in the eight studies that broke down their data by sex (Brosch et al., 1999; Cavenagh et al., 2015; Jenson et al., 2018; Max & Yudman, 2003; Mohammadi & Papadatou-Pastou, 2020; Olander et al., 2010; Records et al., 1977; Rogic-Vidakovic et al., 2016), no sex differences were found, *Q*(1) = 1.49, *p* = 0.22.

#### Small Study Bias

Neither the inspection of the funnel plot (Suppl. Fig. 20) nor Egger’s test (intercept = 0.43 [95% CI:−0.16; 1.02], *t* = 1.41, *p* = 0.16) suggested a small study bias. Eight studies were added using the Trim-and-Fill method to make the funnel plot symmetrical, and no evidence of a difference in ORs was found (OR = 1.11 [95% CI: 0.83; 1.49], *t* = 0.73, *p* = 0.47).

#### Sensitivity Analysis

Bryngelson (1940) and Chrysanthis (1947) were identified both as outliers and influential studies (Suppl. Figs. 21, 22, 23 and 24). Without these two studies, no evidence of a difference in ORs was found (OR = 1.12 [95% CI: 0.92; 1.35], *t* = 1.18, *p* = 0.24). Moreover, no heterogeneity remained, *Q*(42) = 39.20, *p* = 0.59, with no inconsistency between studies, *I*^2^ = 0.00% [95% CI: 0.00%; 35.10%]. The between-study heterogeneity variance was τ^2^ = 0.00 [95% CI: 0.00; 0.18], suggesting a 95% prediction interval from 0.92 to 1.36 around the mean effect, that is including 1 (no difference). Overall, there is evidence that differences exist in the prevalence of non-right-handedness between the two groups, albeit when the sensitivity analysis and the prediction intervals are taken into consideration, this conclusion is not supported.

#### Moderating Variables Analysis

Although we preregistered that should heterogeneity be found we would search for moderating variables, it was clear from the sensitivity analysis that all the heterogeneity was due to the Bryngelson (1940) and the Chrysanthis (1947) studies, which were clear outliers. We therefore did not proceed to a moderating variables analysis.

### Continuous Data Meta-Analysis

**Fig. 6:**
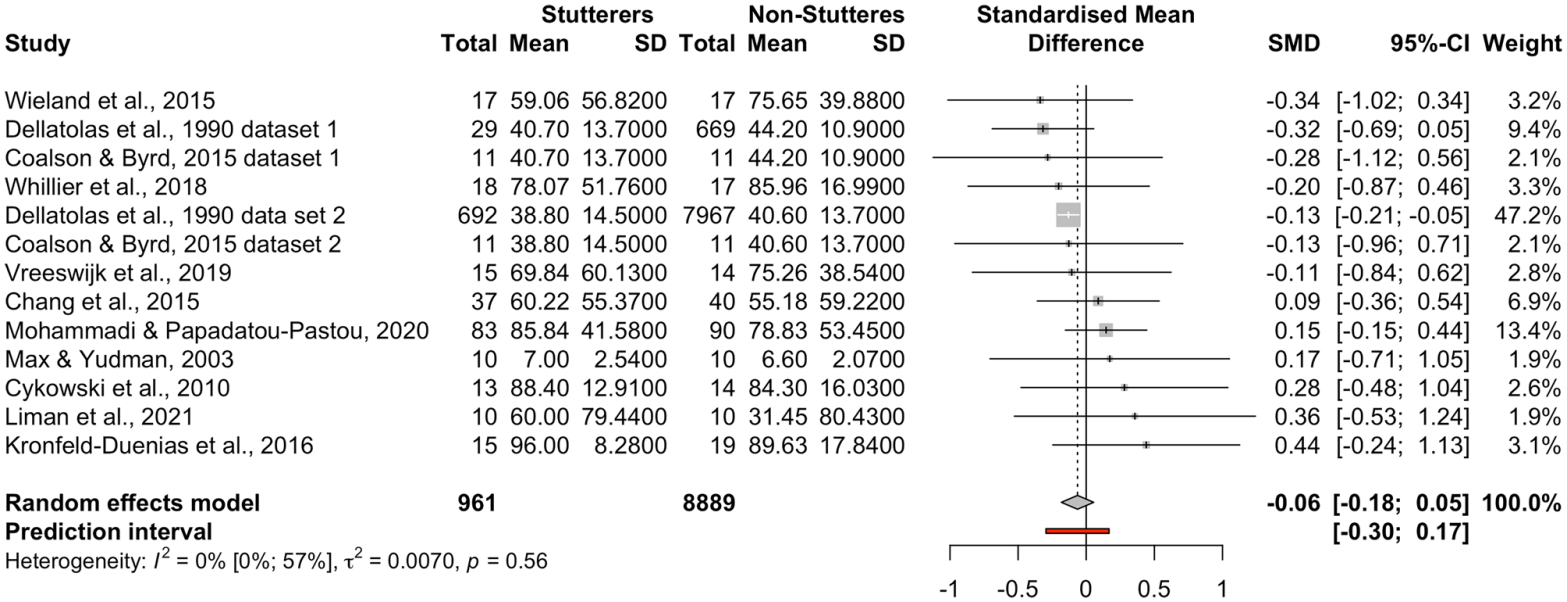
Forest plot of the standardized difference in mean handedness scores between individuals who stutter and individuals-who-do-not-stutter

This meta-analysis included *k* = 13 datasets (from *k* = 11 studies) adding up to *n* = 9850 participants (*n* = 961 individuals who stutter, *n* = 8889 control individuals). Only one study (Dellatolas et al., 1990, study 2) appears to have a significant effect size (*p* < 0.01). The pooled standardized difference in means, *d* = −0.06 [95% CI:−0.18; 0.05],* t* = −1.18, *p* = 0.26 (see forest plot, Fig. 6, and drapery plot, Suppl. Fig. 25). Therefore, there is no evidence that individuals who stutter have lower scores compared to control participants when handedness is measured as a continuous variable. No heterogeneity among the datasets was detected, *Q*(12) = 10.66, *p* = 0.56, with no inconsistency between studies, *I*^2^ = 0.00% [95% CI: 0.00%; 56.60%]. The between-study heterogeneity variance was τ^2^ = 0.01 [95% CI: 0.00; 0.08], suggesting a 95% prediction interval from −0.30 to 0.17 around the mean effect. When comparing males and females in the three studies that broke down their data by sex (Kronfeld-Duenis et al., 2016; Max & Yudman, 2003; Mohammadi & Papadatou-Pastou, 2020), no sex differences were found, *Q*(1) = 0.00, *p* = 0.96.

#### Small Study Bias

Neither the inspection of the funnel plot (Suppl. Fig. 26) nor Egger’s test (intercept = 0.46 [95% CI:−0.18; 1.1], *t* = 1.42, *p* = 0.18) suggested a small study bias. Three studies were added using the Trim-and-Fill method to make the funnel plot symmetrical, but again, no evidence of a difference was found (*d* = −0.11 [95% CI:−0.20; 0.02], *t* = −2.68, *p* = 0.02).

#### Sensitivity Analysis

No outliers were identified, which was expected as no heterogeneity was found. The second dataset by Dellatollas et al. (1990) appears influential in the influence plots (Suppl. Figs. 27, 28, 29 and 30). It further carries 47.2% of the weight of the pooled analysis. We therefore repeated the meta-analysis by removing this study. Now, the meta-analysis included *k* = 12 datasets (from 11 studies) adding up to *n* = 1191 participants (*n* = 269 individuals who stutter, *n* = 922 control individuals). The pooled standardized difference in means was *d* = −0.004 [95% CI:−0.17; 0.16],* t* = −0.06, *p* = 0.95. Again, there is no evidence of a handedness difference between individuals who stutter and control participants. Heterogeneity among the datasets was not detected again, *Q*(11) = 8.66, *p* = 0.65, with no inconsistency between studies, *I*^2^ = 0.00% [95% CI: 0.0%; 58.3%]. The between-study heterogeneity variance was τ^2^ = 0.01 [95% CI: 0.00; 0.10], suggesting a 95% prediction interval from−0.27 to 0.26 around the mean effect (i.e., including zero).

## Discussion

It was the aim of the present study to evaluate whether a link between atypical handedness and stuttering exists, by addressing handedness differences between individuals who stutter compared to individuals who do not stutter (controls). To that end, five separate meta-analyses were conducted on studies measuring handedness in *n* = 19,738 participants (*n* = 2590 individuals who stutter, *n* = 17,148 controls). The first four meta-analyses concerned categorical classifications of handedness and used the odds ratio as the effect size [left-handers (forced choice); left-handers (extreme); mixed-handers; non-right-handers vs. total)] and the fifth one concerned continuous handedness data and used the standardized difference in means as the effect size. Overall, findings are inconclusive regarding the presence of a link between atypical handedness and stuttering.

Specifically, no evidence that individuals who stutter have a higher prevalence of left-handedness (extreme) or mixed-handedness compared to controls (*p* = 0.49 and *p* = 0.74, respectively) was found. Additionally, the analysis revealed that individuals who stutter have similar scores compared to control participants when handedness is measured as a continuous variable (*p* = 0.26). However, evidence that individuals who stutter have higher prevalence of left-handedness (forced choice) and non-right-handedness compared to controls did emerge (*p* = 0.01 and *p* = 0.007, respectively). Yet, in the latter case, the prediction interval around the mean effect—the range into which we can expect the effects of future studies to fall based on present evidence—included an odds ratio value of 1.0 (prediction interval: 0.71 to 2.80), which corresponds to the null hypothesis of no handedness differences between the two groups. Moreover, when sensitivity analysis was performed by removing the two studies that were identified as both outliers and influential studies, no evidence of a difference remained (*p* = 0.23). Thus, the evidence of a difference that emerged for the non-right-handedness meta-analyses should be treated with caution. In the case of the left-handedness (forced-choice) meta-analysis, the lower bound of the prediction interval was close to an odds ratio value of 1.0 (prediction interval: 1.01 to 2.42), which might again be taken into consideration when interpreting findings. Overall, the present findings do not allow for strong conclusions to be made with regard to the relationship between atypical handedness and stuttering. What we suggest is that a relationship, should it exist, is weak at best.

One could interpret the fact that only the left-handedness (forced choice) and the non-right-handedness meta-analyses showed evidence of a relationship between stuttering and atypical handedness as a matter of statistical power; should one order the meta-analyses according to the number of the included data sets (*k* = 13, *k* = 16, *k* = 16, *k* = 24, *k* = 45), then the corresponding *p* values would be *p* = 0.26, *p* = 0.49,* p* = 0.74,* p* = 0.01, *p* = 0.007, suggesting that only larger meta-analyses are providing evidence of a difference. However, when removing two studies in the largest meta-analysis (the non-right-handedness meta-analysis) for the purposes of the sensitivity analysis, then there was no longer evidence of a difference. Thus, statistical power seems to not be an adequate explanation for the present findings.

We put forward another interpretation, that the fact that only left-handedness (forced choice) and the non-right-handedness meta-analyses showed evidence of a relationship between stuttering and atypical handedness is a consequence of the nature of the forced choice classification itself (which was also included in the non-right-handedness comparison, which included studies that used both the left-handedness (forced choice) and left-handedness (extreme) classifications). In studies where participants are forced to choose between declaring that they are left- or right-handed (or they are grouped in these categories by the researchers after they have completed some kind of handedness assessment), the participants who naturally fall at the middle of the handedness continuum (those with weak preference or those who are mixed-handed, or ambidextrous) are lumped together with either left- or right-handers. The criteria for classification into different handedness groups moreover differ between studies. Of note, this “middle” category is actually quite large, corresponding to 9.33% of the general population (95% CI: 6.67%, 12.00%) as shown by a recent large-scale meta-analysis (Papadatou-Pastou et al., 2020). In other words, this “middle” category concerns as many individuals as those that are left-handed using stringent criteria (9.34%, 95% CI: 7.92%, 10.80%; Papadatou-Pastou et al., 2020). What is more is that when participants are classified into left- or right-handers using hand preference questionnaires (with a cut-off score at the middle of the continuum) compared to when they are self-classified or when writing hand is used as the handedness criterion, a mismatch of only 0.4% takes place for right-handers, when this mismatch reaches 13.5% in the case of left-handers (Papadatou-Pastou et al., 2013). It is probably the middle category that is being mismatched and which is misplaced in the cases of forced two-way classifications. Papadatou-Pastou et al. (2020) have suggested that capturing mixed-handedness might improve the power to address questions within handedness research. Taking all these together, we suggest that the left-handedness (forced-choice) category is not clearly defined and thus not very informative when it comes to the relationship of handedness with different conditions, such as stuttering.

In terms of moderators, only the mixed-handedness meta-analysis was found to exhibit heterogeneity after the sensitivity analysis, thus only for that meta-analysis was the presence of moderators explored, as preregistered. No moderators were detected (among mean age, year of publication, sex ratio, handedness measurement and purpose of the study). However, it must be stressed that the number of studies included in the moderator variables analysis was very small; therefore, the power of this analysis to detect any relationship was low. No sex differences were further found in any of the meta-analyses when directly comparing the two sexes, but these comparisons were again based on the very limited number of studies that broke down their data by sex. Other variables with a possible moderating effect (namely self-report and location) could not be examined within the mixed-handedness meta-analysis due to insufficient data.

It should be noted that the studies included in the analyses assessed handedness in different ways (EHI, drawing, parental-reported, single dimension “are you a right/left-hander?”). This difference in assessing handedness can influence the results (see mismatch between self-report and hand preference questionnaires discussed above), but not the base rate of handedness in each study. Moreover, some studies did not report how hand preference was scored and/or how being left- or right-handed was categorized (e.g., Bryngelson & Rutherford, 1937; Bryngelson, 1940; Maxfield et al., 2015). Additionally, the included studies have a great variety in the mean age of participants (ranging from 4 to 35 years of age), which could have influenced the overall results. The study by Brosch et al. (1999) indicated that left-handed children had a poorer chance of attaining speech fluency when compared to right-handed stutterers. Thus, to investigate a link between stuttering and hand preference, it would have been interesting to further disentangle a correlation between the severity of stuttering and hand preference. However, the severity of stuttering could not be used as a moderator given that only three papers (Maruthy et al., 2017; Olander et al., 2010; Rogić Vidaković, 2016) gave enough information to allow for different severities of stuttering to be compared within the handedness categories. Yet, heterogeneity failed to reach significance levels in any meta-analyses other than the mixed-handedness one, making it unlikely that there is a subgroup of studies showing a result pattern differing from the main result.

Another speech fluency disorder, for which (non-significantly) elevated levels of non-right-handedness have been reported (Howell & Davis, 2011), is cluttering. Cluttering is characterized by speech that is perceived as too rapid and/or irregular, and/or with irregularly occurring phonetic/phonological abnormalities, contraction or omission of syllables, abnormal pauses, syllable stress, and speech rhythm, as well as dysfluencies that are atypical for stuttering (Neumann et al., 2017). We did not include cluttering in our meta-analysis, because it remains unclear whether these two disorders are related or not (for a discussion see Howell & Davis, 2011).

This study holds limitations that are important to consider for interpreting the results. First of all, the analysis only included studies on hand preference but not on hand skill, which is another important manifestation of handedness. Indeed, hand preference and hand skill have been suggested to be independently lateralized (Triggs et al., 2000), while the correlation between preference and skill depends on which tests are used to assess these variables (Buenaventura Castillo et al., 2020). Thus, no firm conclusion can be drawn on hand skill and stuttering from the present analyses. Furthermore, the meta-analysis only investigated the direction (e.g., left- vs. right-handedness) but not the strength (degree) of hand preference (e.g., weak vs. strong handedness), although the continuous data meta-analysis could be informative for strength of hand preference. This is another important distinction, as some specific genetic polymorphisms, such as the *PCSK6* gene, have been associated with degree but not direction of hand preference (Arning et al., 2013). Degree might actually be a more powerful and suitable way for classification of handedness than direction (for a review see Prichard et al., 2013). Hand skill and strength of handedness were not considered, as no studies reporting information on these manifestations of handedness were located through our search (Liman et al., 2021, measured the number of finger taps with both thumbs, but only report the findings in figures).

Another limitation is the fact that mixed-handedness was equated to a middle category for the purposes of the meta-analysis. Yet, this middle category is defined differently in different studies (e.g., no/weak preference, mixed-handedness ambidexterity, latent left-handedness). It is important to clarify that these definitions are not interchangeable; for example, mixed-handers use different hands for different activities, while ambidextrous people (no preference) might use different hands for the same activity at different occasions. Additionally, as also mentioned above, the data were not broken down by sex or by severity of stuttering in most studies, to allow for meaningful comparisons. In terms of age, studies reported data on children and young adults up to 35 years of age, therefore no data were available on older adults to allow for developmental effects beyond these ages to be investigated. Lastly, Harrer et al. (2019) suggested a limit of 10 studies for performing a meta-analysis. This limit is almost reached in the meta-analyses for mixed-handedness (*n* = 14) and left-handedness (extreme) (*n* = 14). As far as the reviewing process itself is concerned, as the literature on stuttering and handedness spans decades, we could not get in contact with the authors of older papers to ask for clarifications or data that were not reported in the papers.

More empirical studies and updated meta-analyses on the relationship of atypical handedness and stuttering are needed to draw any firm conclusions, as the present set of meta-analyses of available evidence did not provide robust evidence of a relationship or lack thereof. Future studies on stuttering and handedness would benefit from reporting handedness data on both hand preference and hand skill as well as on handedness direction and handedness strength. Moreover, mixed-handedness should be reported as a separate category. Data should also be broken down by severity of stuttering. We further join recently voiced recommendations of handedness meta-analyses (e.g., Nastou et al., 2022; Packheiser et al., 2020; Papadatou-Pastou et al., 2020) for uploading raw data in open access repositories, such as osf.io, so that future meta-analysts can have access to these data.

The weak, if existing, relationship between handedness and atypical handedness and stuttering, allow us to make recommendations for clinical practice as well as for educators. In both cases, atypical handedness should not be treated as a central risk factor for stuttering, although a note could be made to allow for this information to be assessed in the context of a clinical diagnosis. Similarly, parents should not be alerted to the fact that their child is not right-handed in the context of stuttering signs.

## Conclusion

The present study was a meta-analytical synthesis of all available evidence on the relationship between stuttering and hand preference. Five separate meta-analyses were conducted that correspond to different conceptualizations of atypical hand preference [left-handers (forced choice); left-handers (extreme); mixed-handers; non-right-handers vs. total)] as well as continuous handedness. No evidence of a link between atypical handedness and stuttering was found for the left-handedness (extreme), mixed-handedness and continuous data meta-analyses. However, evidence did emerge for the left-handedness (forced choice) and the non-right-handedness meta-analyses. Therefore, the evidence for the relationship between stuttering and atypical handedness are at this point inconclusive. We suggest that a relationship, should future meta-analyses show that it exists, is weak at best. This is in contrast to other neurodevelopmental disorders, such as autism spectrum disorder, for which strong evidence of a relationship with atypical handedness is reported (Markou et al., 2017). Therefore, a disorder-specific approach is important when investigating handedness differences in different neurodevelopmental and psychiatric disorders, as previously suggested (Mundorf & Ocklenburg, 2021; Nastou et al., 2022).

## Supplementary Information

Below is the link to the electronic supplementary material.

Supplementary file1 (PNG 115 KB)

Supplementary file2 (PNG 1240 KB)

Supplementary file3 (PNG 37 KB)

Supplementary file4 (PNG 194 KB)

Supplementary file5 (PNG 193 KB)

Supplementary file6 (PNG 275 KB)

Supplementary file7 (PNG 827 KB)

Supplementary file8 (PNG 328 KB)

Supplementary file9 (PNG 178 KB)

Supplementary file10 (PNG 98 KB)

Supplementary file11 (PNG 107 KB)

Supplementary file12 (PNG 98 KB)

Supplementary file13 (PNG 48 KB)

Supplementary file14 (PNG 326 KB)

Supplementary file15 (PNG 184 KB)

Supplementary file16 (PNG 380 KB)

Supplementary file17 (PNG 379 KB)

Supplementary file18 (PNG 1057 KB)

Supplementary file19 (PNG 92 KB)

Supplementary file20 (PNG 52 KB)

Supplementary file21 (PNG 157 KB)

Supplementary file22 (PNG 107 KB)

Supplementary file23 (PNG 870 KB)

Supplementary file24 (PNG 91 KB)

Supplementary file25 (PNG 47 KB)

Supplementary file26 (PNG 105 KB)

Supplementary file27 (PNG 104 KB)

Supplementary file28 (PNG 158 KB)

Supplementary file29 (PNG 1616 KB)

Supplementary file30 (PNG 48 KB)

## Data Availability

Materials (i.e., preregistration, Zotero files before and after duplicates were merged, list of full-texts screened and excluded), data, and analysis code is available in the Open Science Framework, an online open-access repository, at https://osf.io/xse5g/.
