# Supplementary figures and images for: Hand Preference in Stuttering: Meta-Analyses
Source: Neuropsychol Rev. 2023 Oct 5;34(3):924–51. doi: 10.1007/s11065-023-09617-z (PMC11473670; doi:10.1007/s11065-023-09617-z)

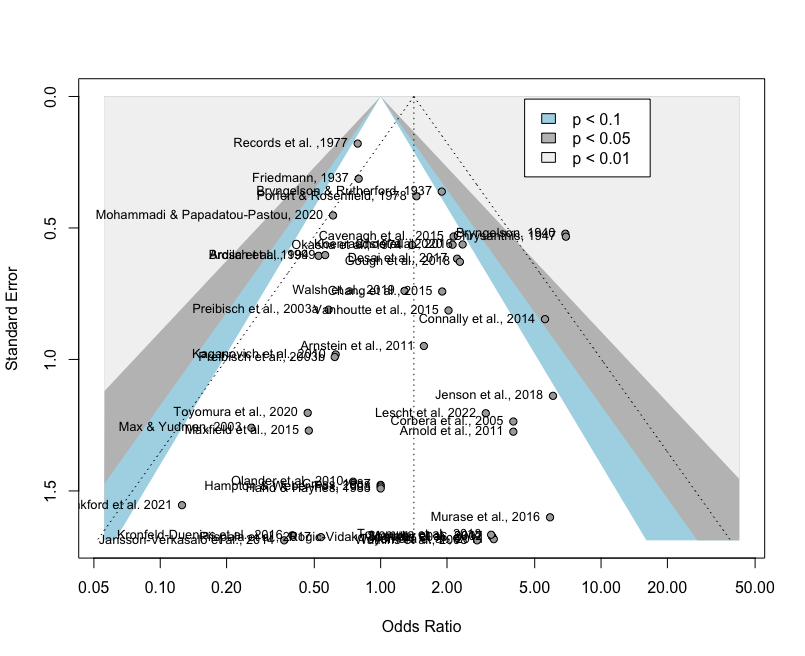

Supplement: Supplementary file 1 — Supplementary file1 (PNG 115 KB) [file 11065_2023_9617_MOESM1_ESM.png]

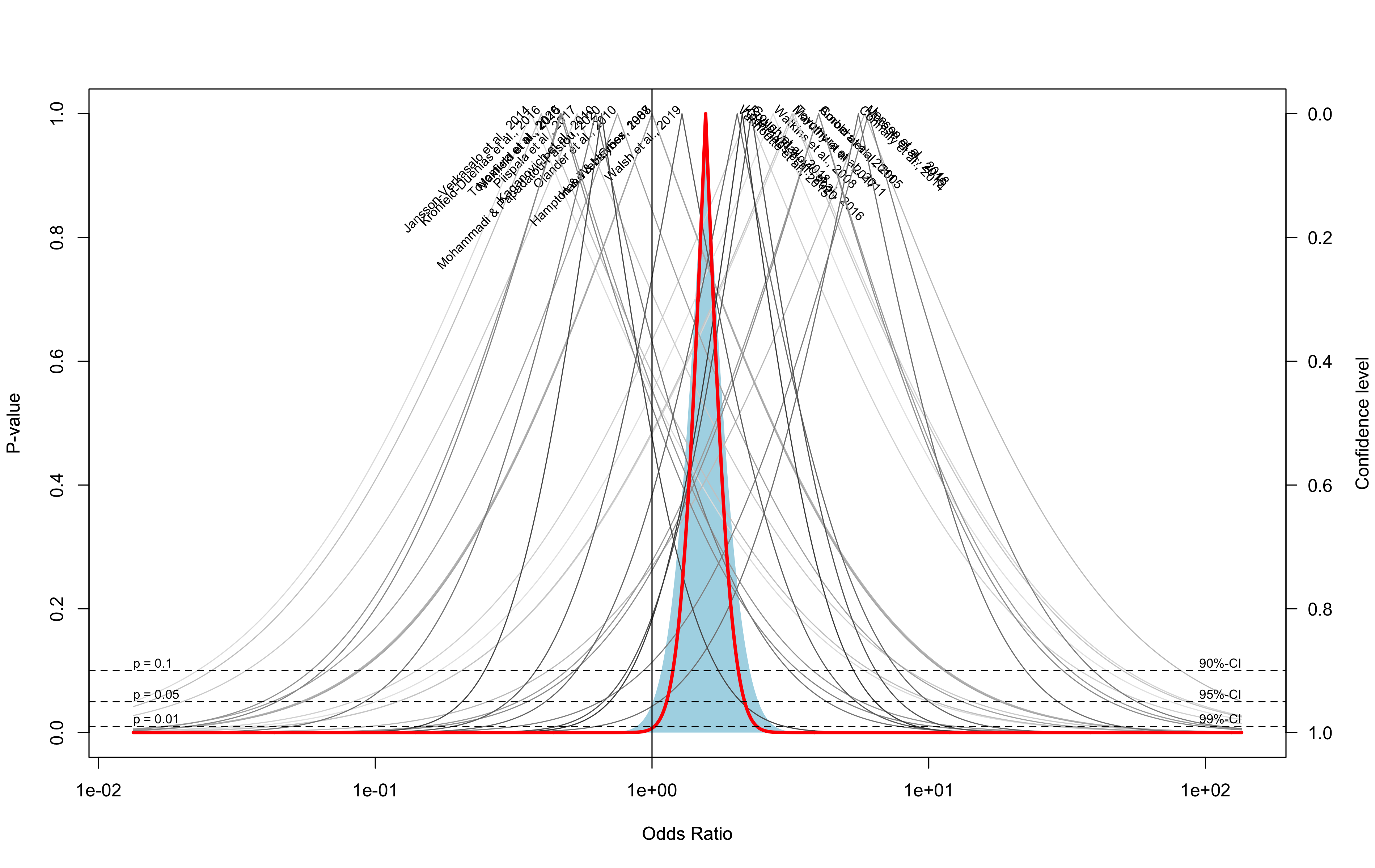

Supplement: Supplementary file 2 — Supplementary file2 (PNG 1240 KB) [file 11065_2023_9617_MOESM2_ESM.png]

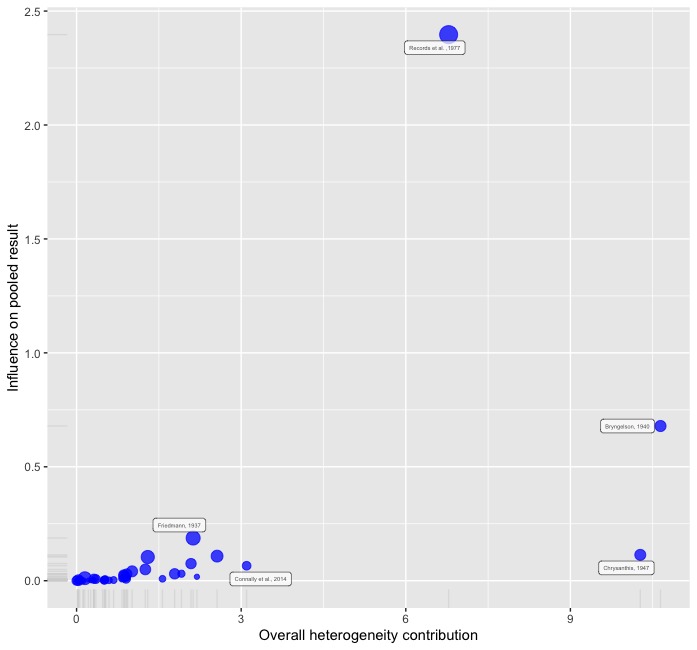

Supplement: Supplementary file 3 — Supplementary file3 (PNG 37 KB) [file 11065_2023_9617_MOESM3_ESM.png]

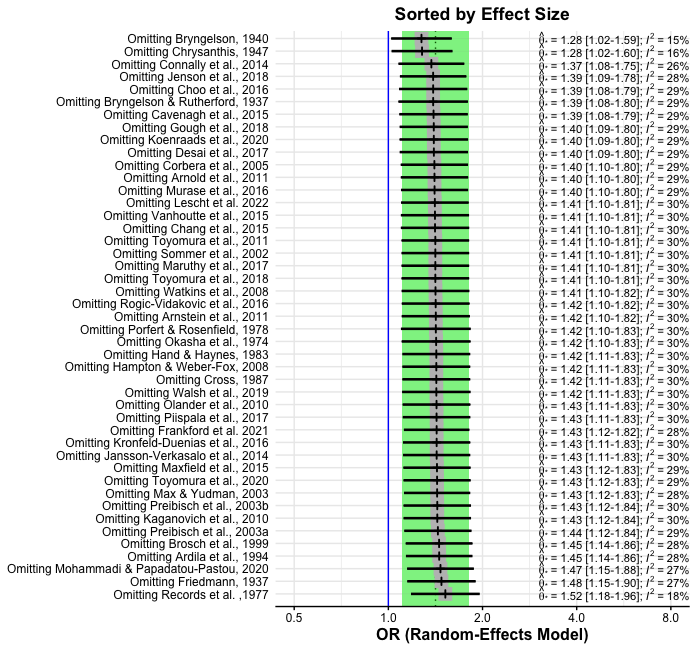

Supplement: Supplementary file 4 — Supplementary file4 (PNG 194 KB) [file 11065_2023_9617_MOESM4_ESM.png]

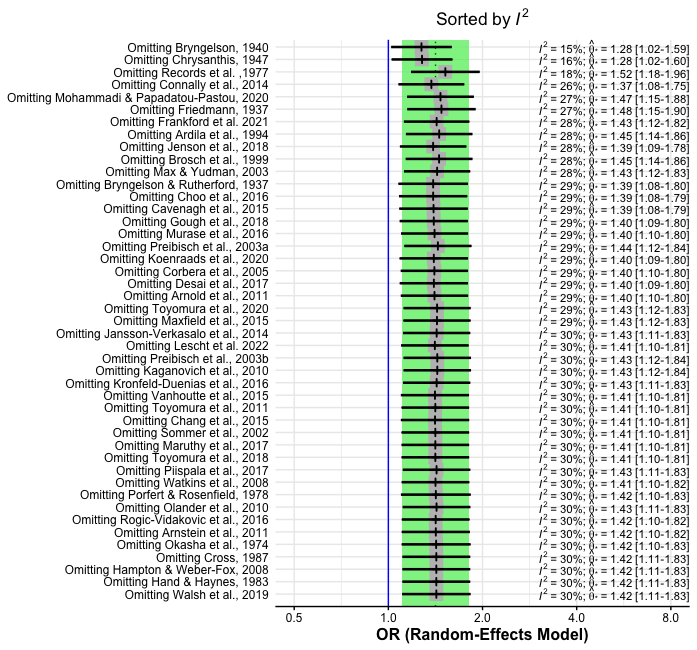

Supplement: Supplementary file 5 — Supplementary file5 (PNG 193 KB) [file 11065_2023_9617_MOESM5_ESM.png]

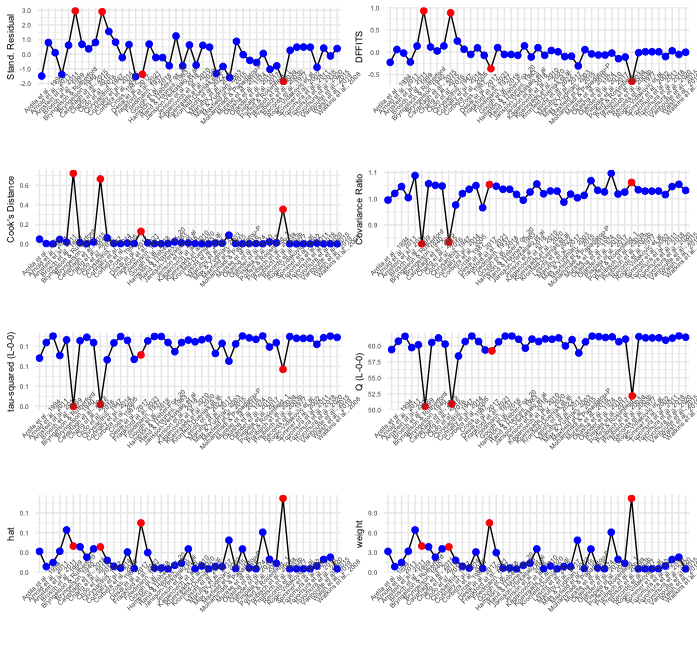

Supplement: Supplementary file 6 — Supplementary file6 (PNG 275 KB) [file 11065_2023_9617_MOESM6_ESM.png]

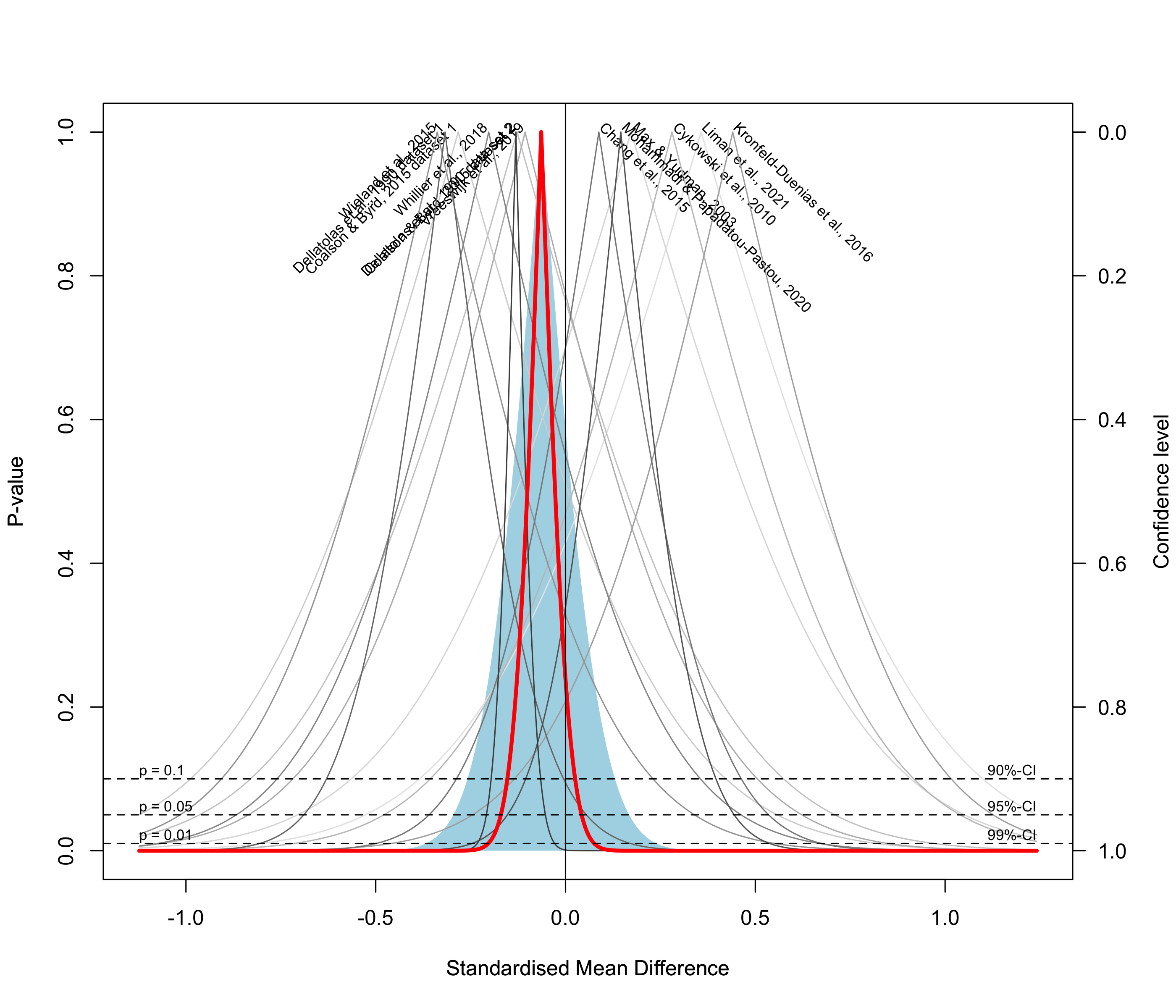

Supplement: Supplementary file 7 — Supplementary file7 (PNG 827 KB) [file 11065_2023_9617_MOESM7_ESM.png]

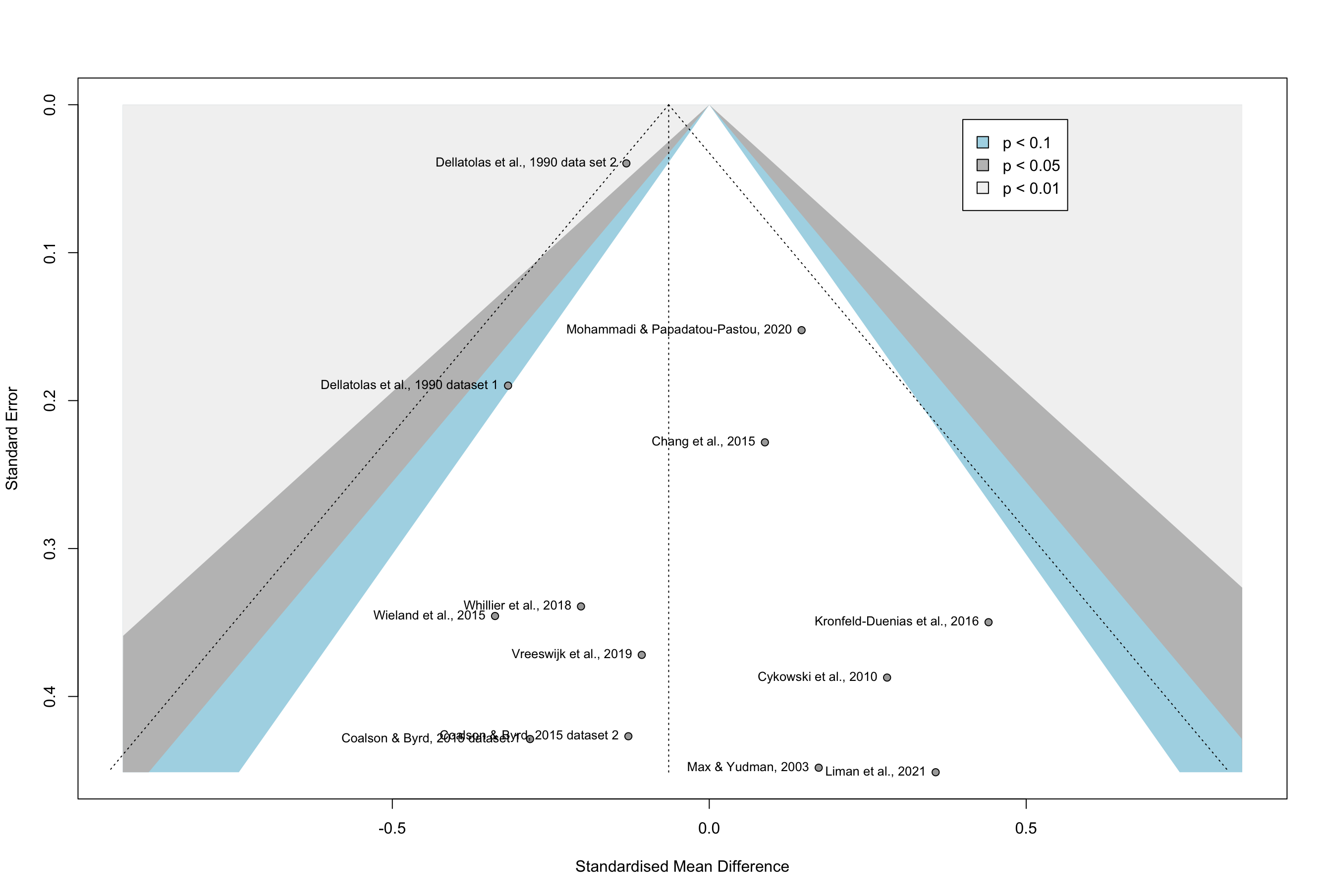

Supplement: Supplementary file 8 — Supplementary file8 (PNG 328 KB) [file 11065_2023_9617_MOESM8_ESM.png]

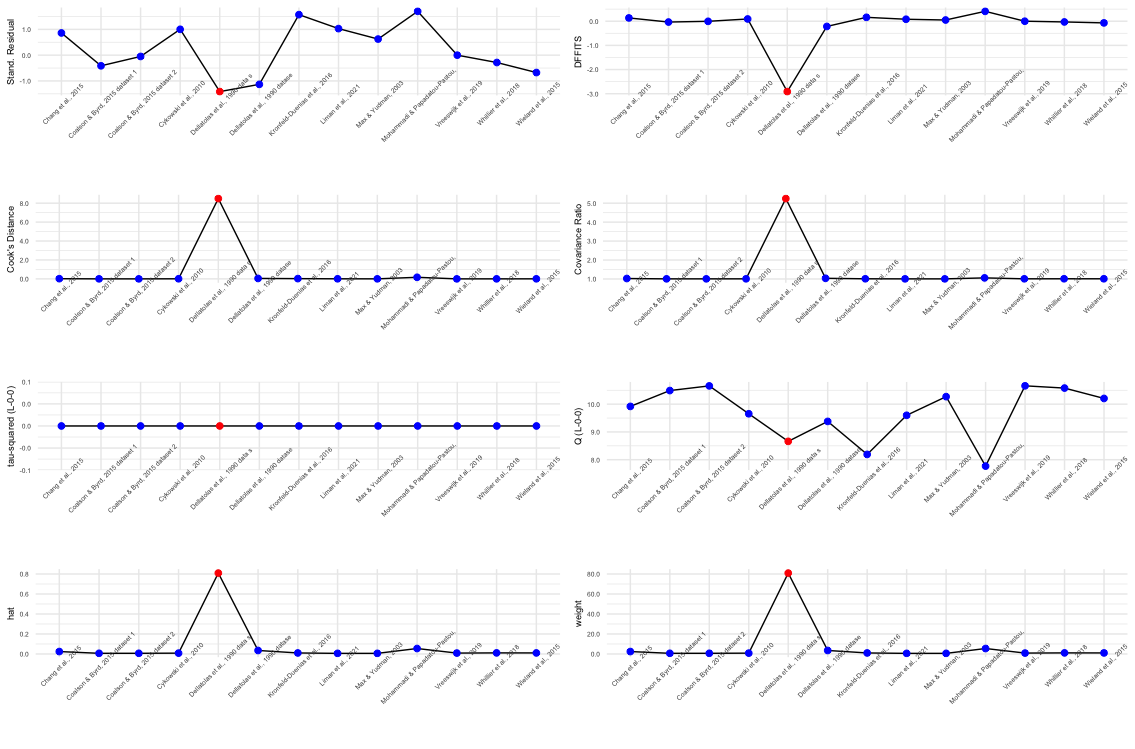

Supplement: Supplementary file 9 — Supplementary file9 (PNG 178 KB) [file 11065_2023_9617_MOESM9_ESM.png]

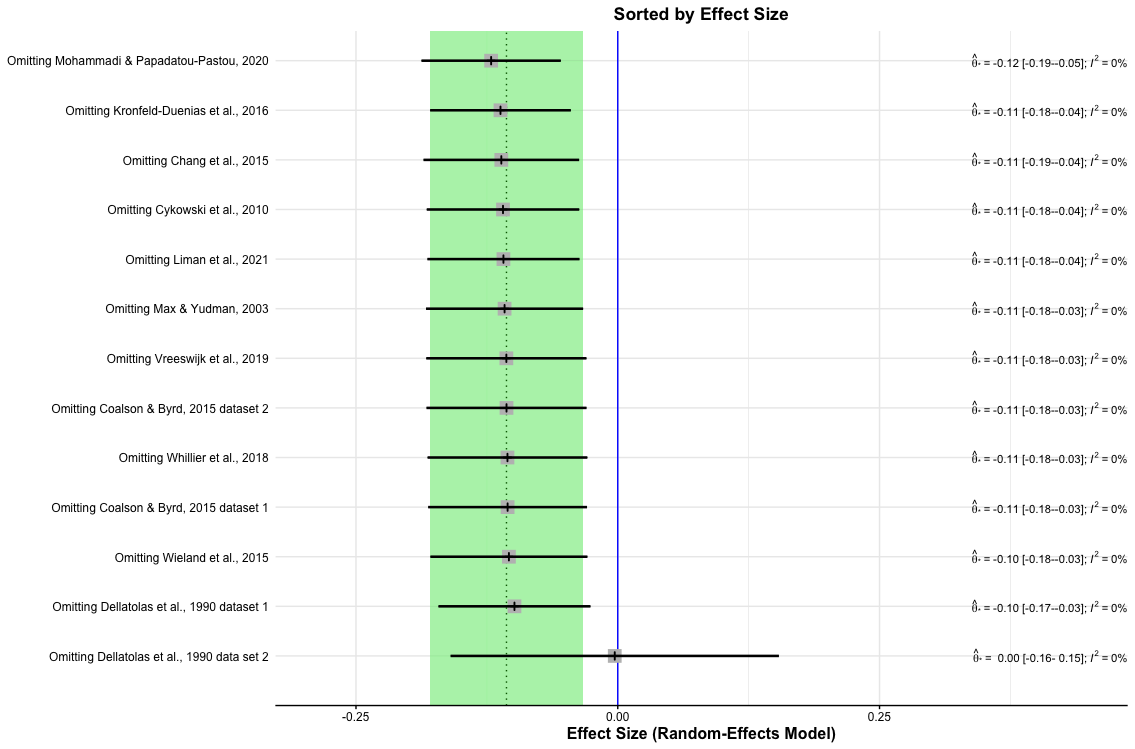

Supplement: Supplementary file 10 — Supplementary file10 (PNG 98 KB) [file 11065_2023_9617_MOESM10_ESM.png]

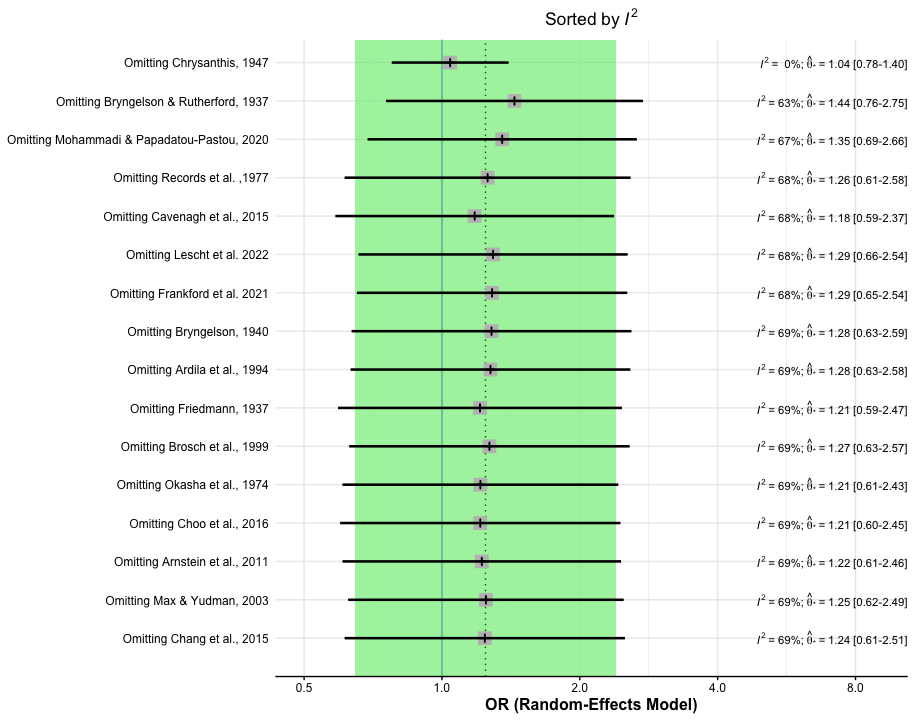

Supplement: Supplementary file 11 — Supplementary file11 (PNG 107 KB) [file 11065_2023_9617_MOESM11_ESM.png]

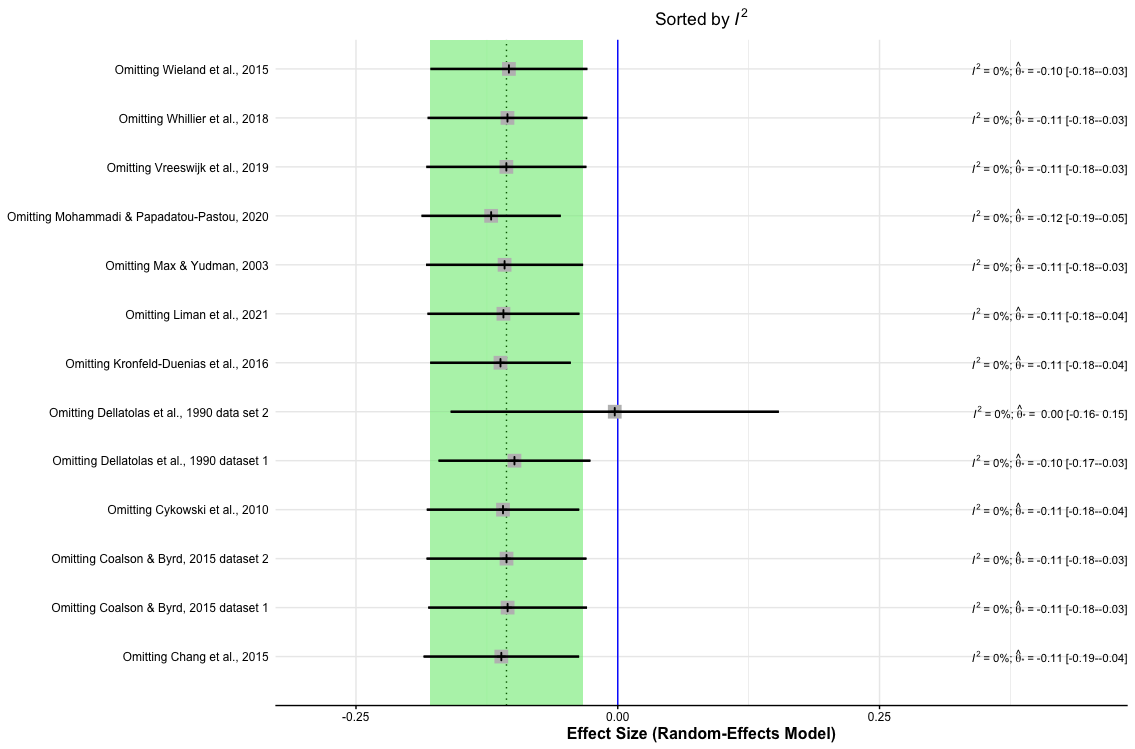

Supplement: Supplementary file 12 — Supplementary file12 (PNG 98 KB) [file 11065_2023_9617_MOESM12_ESM.png]

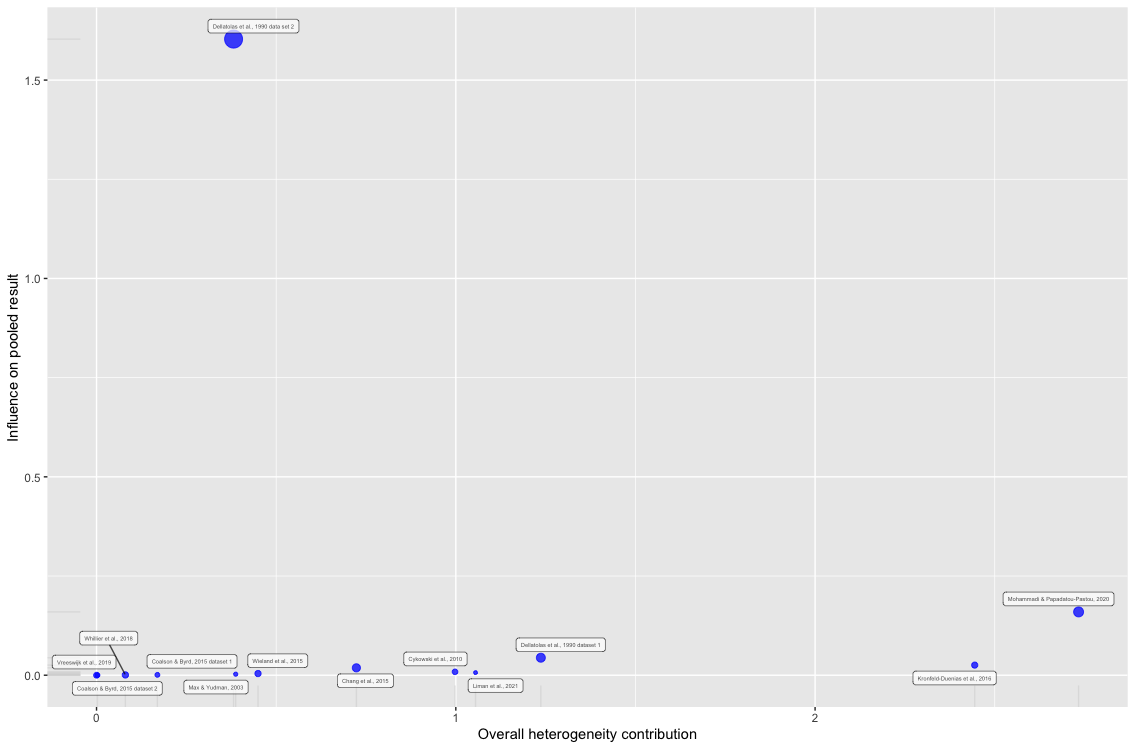

Supplement: Supplementary file 13 — Supplementary file13 (PNG 48 KB) [file 11065_2023_9617_MOESM13_ESM.png]

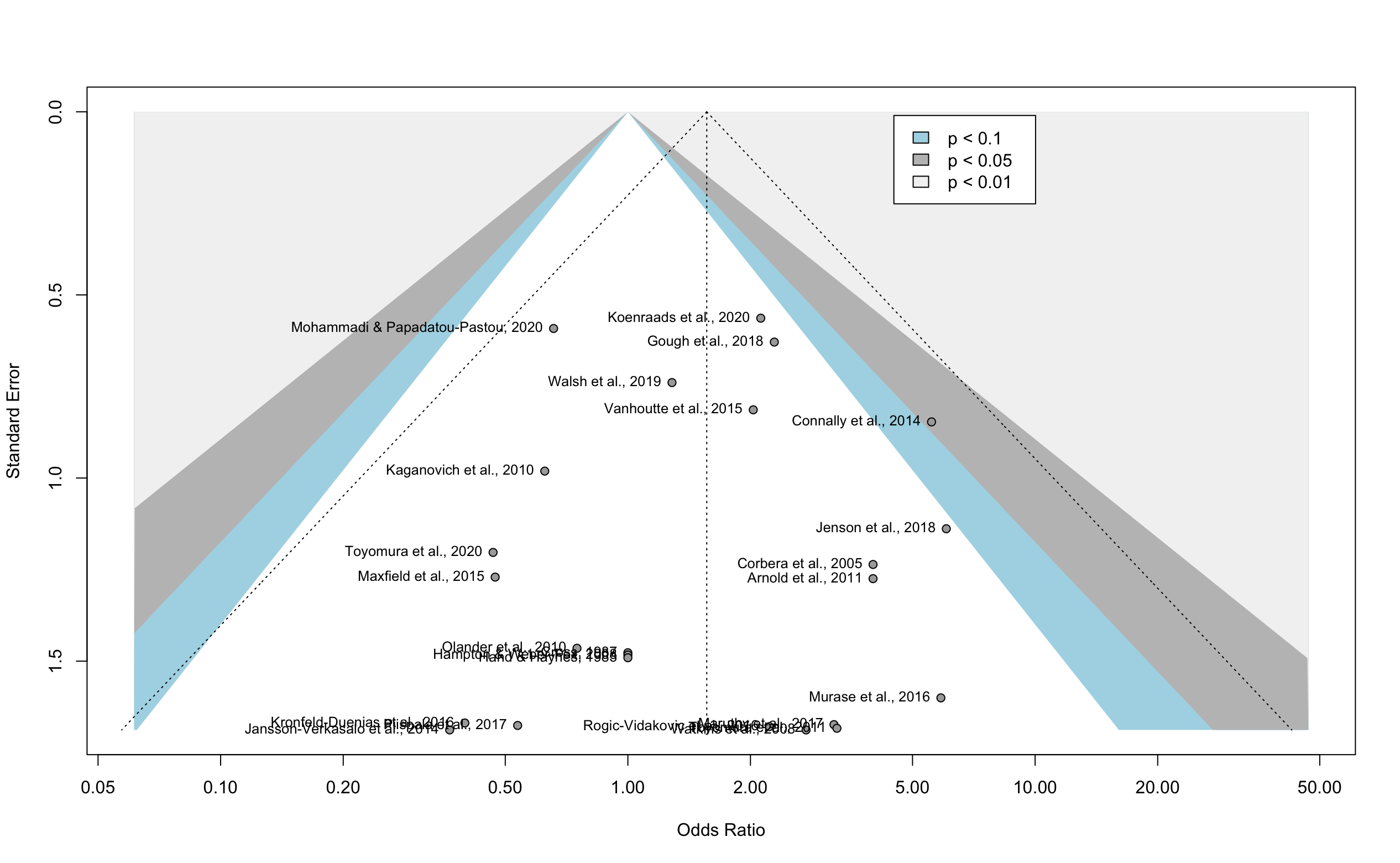

Supplement: Supplementary file 14 — Supplementary file14 (PNG 326 KB) [file 11065_2023_9617_MOESM14_ESM.png]

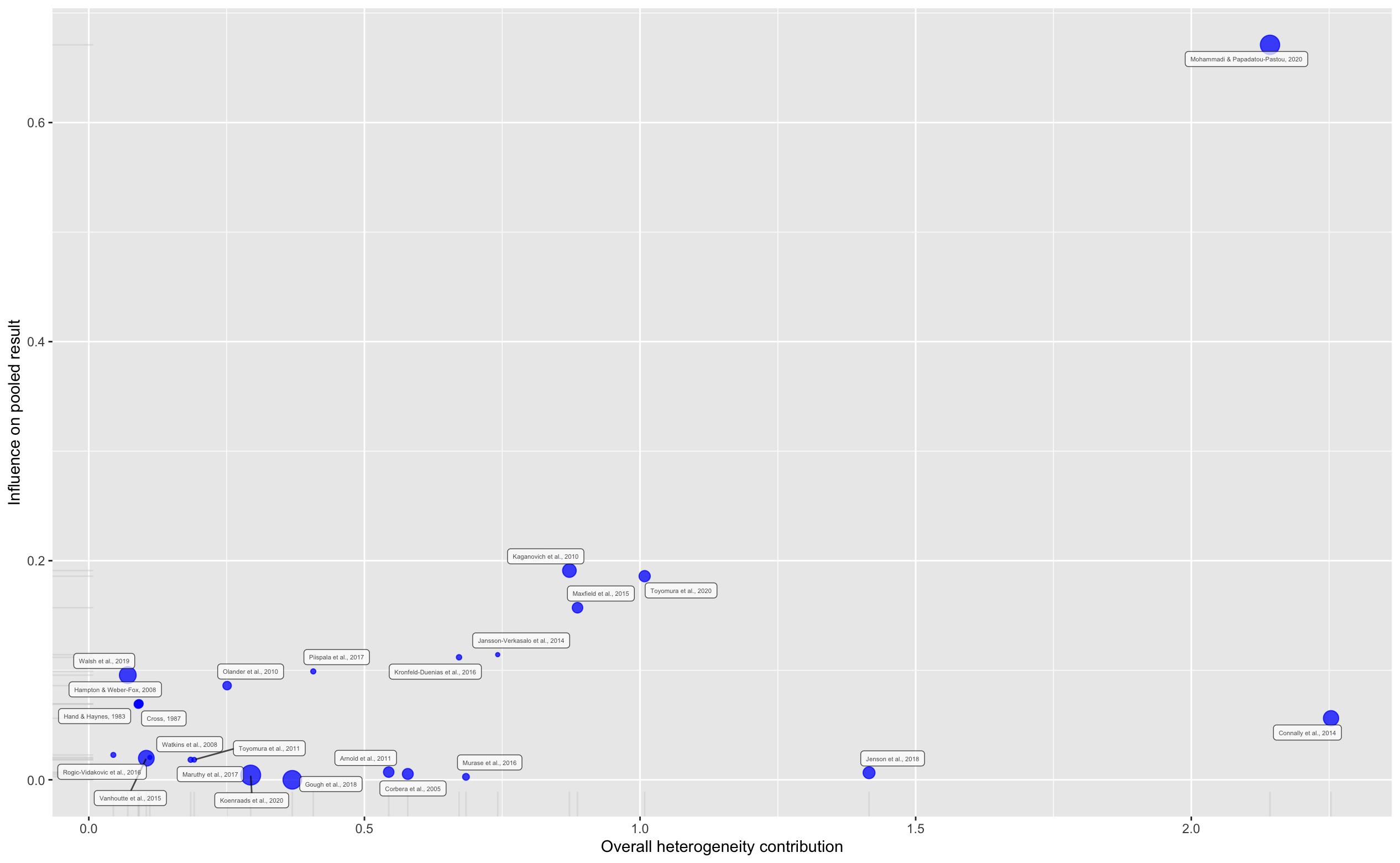

Supplement: Supplementary file 15 — Supplementary file15 (PNG 184 KB) [file 11065_2023_9617_MOESM15_ESM.png]

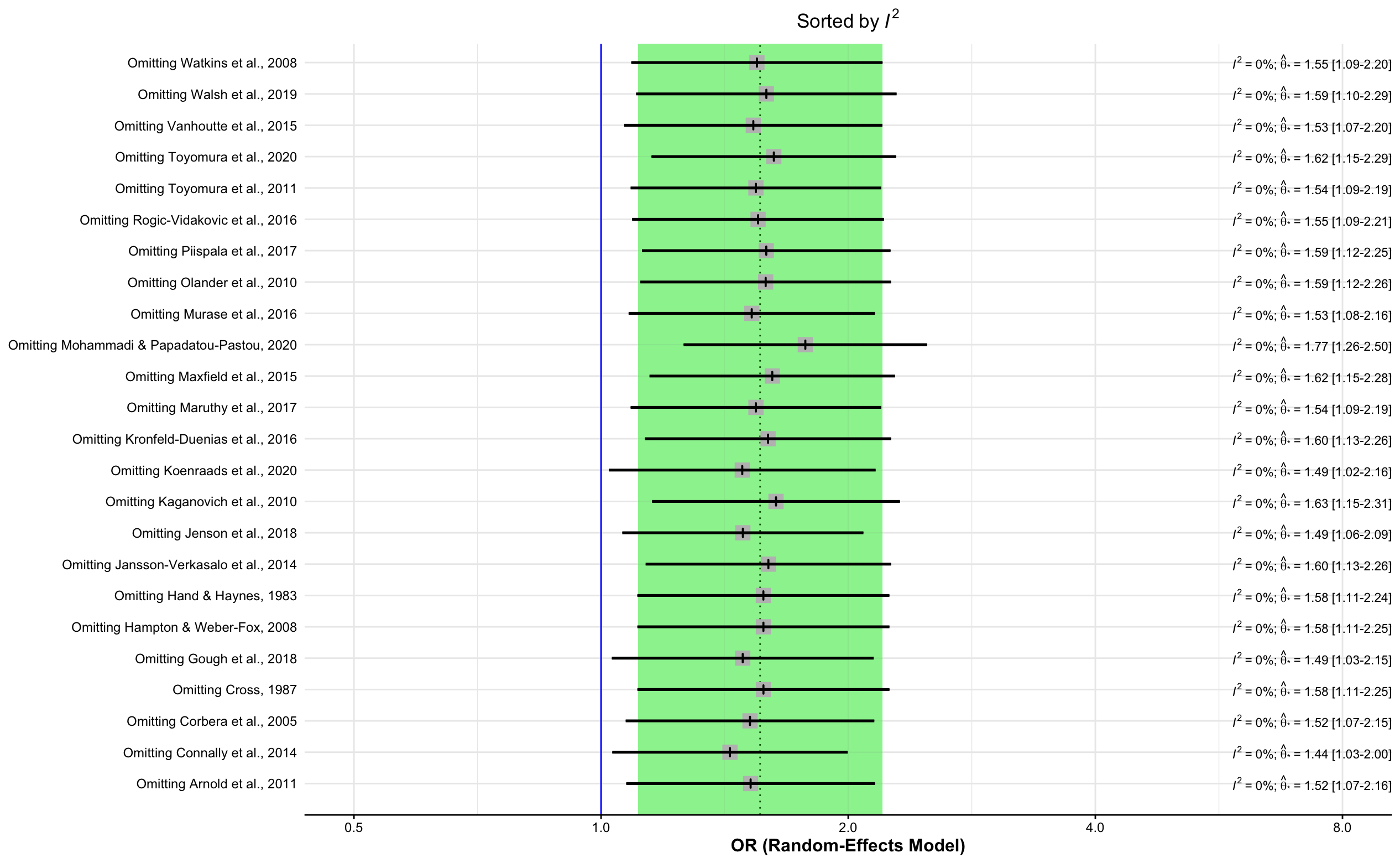

Supplement: Supplementary file 16 — Supplementary file16 (PNG 380 KB) [file 11065_2023_9617_MOESM16_ESM.png]

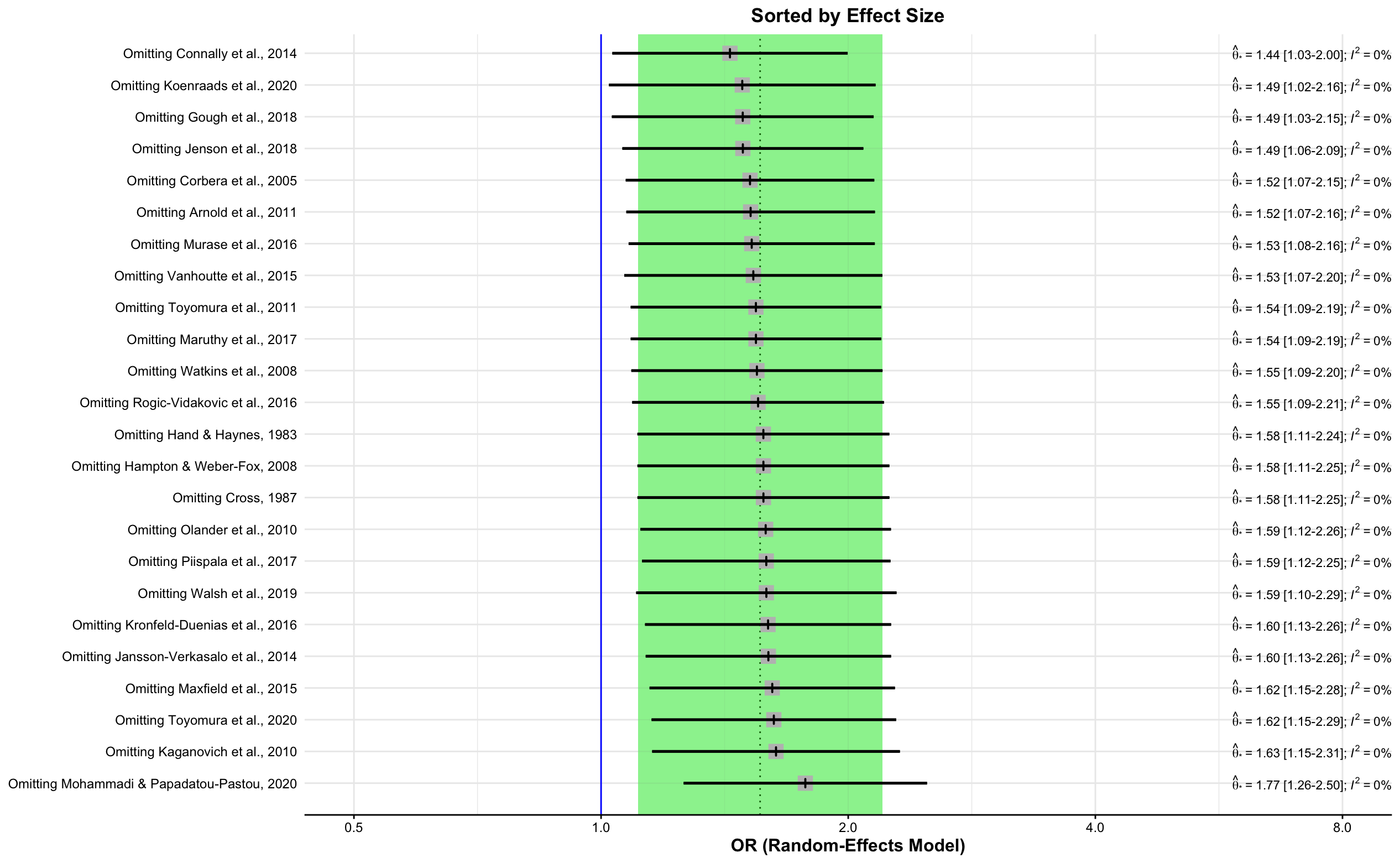

Supplement: Supplementary file 17 — Supplementary file17 (PNG 379 KB) [file 11065_2023_9617_MOESM17_ESM.png]

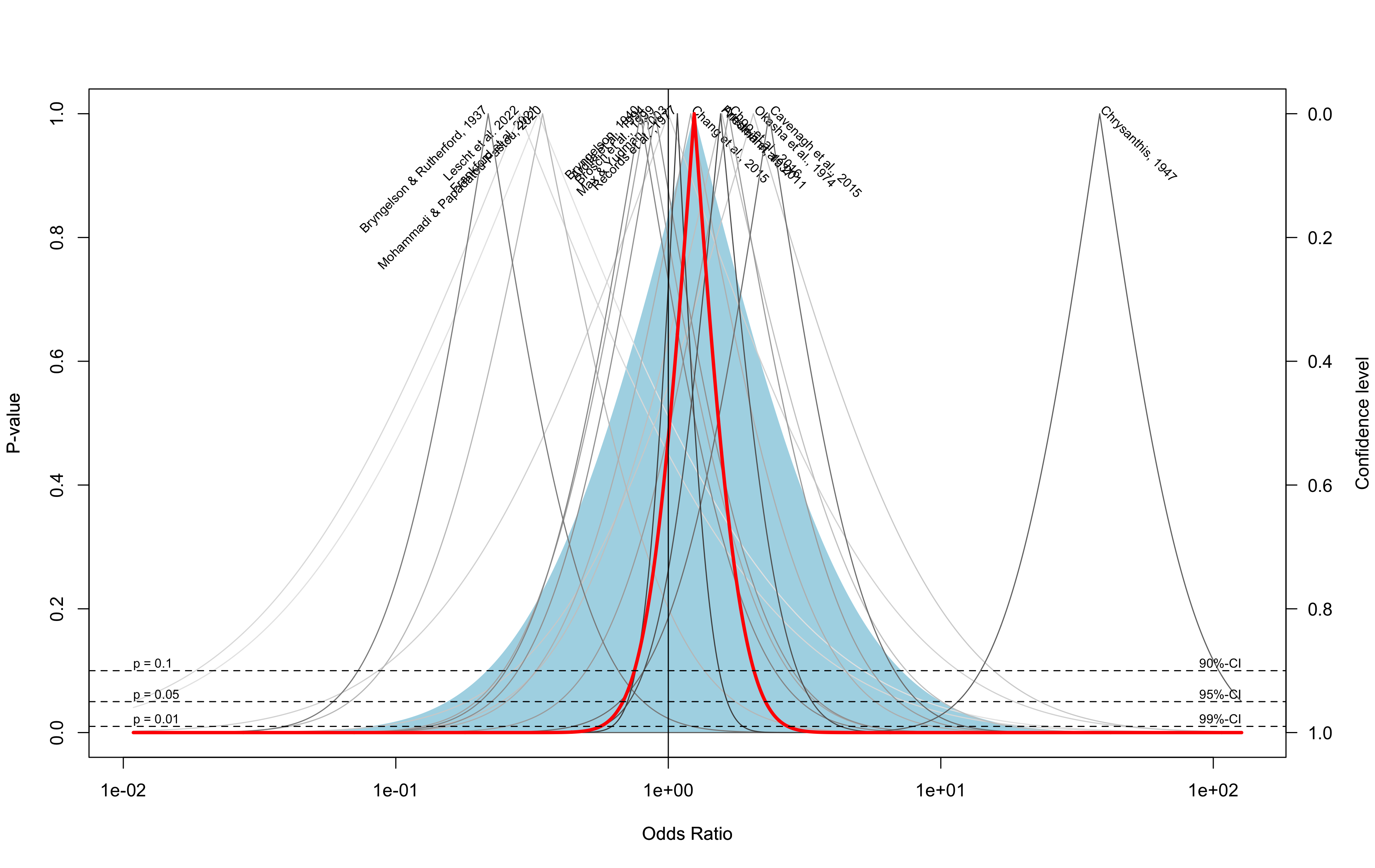

Supplement: Supplementary file 18 — Supplementary file18 (PNG 1057 KB) [file 11065_2023_9617_MOESM18_ESM.png]

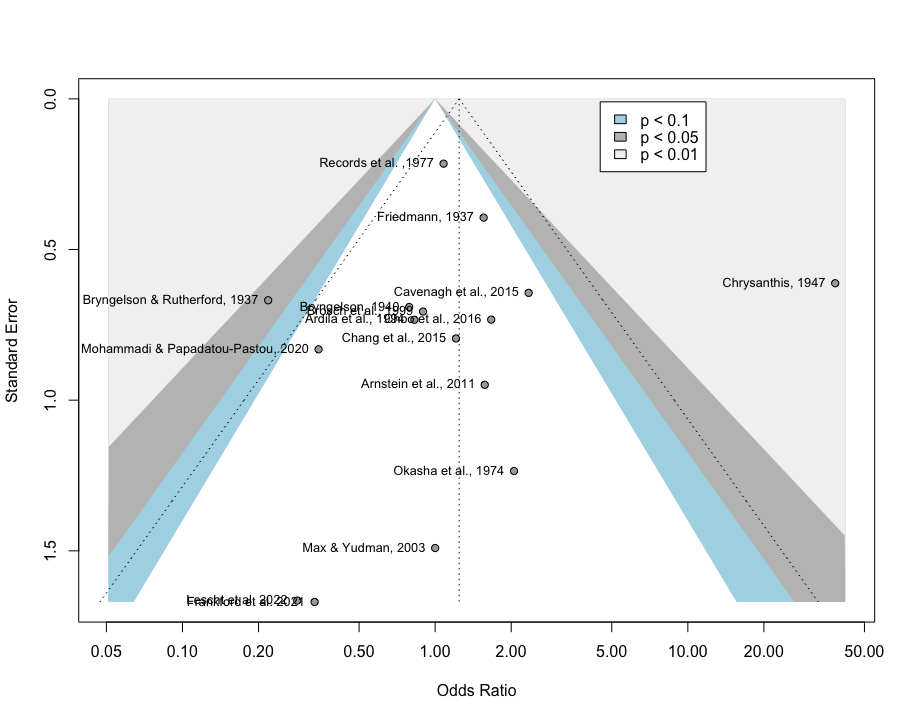

Supplement: Supplementary file 19 — Supplementary file19 (PNG 92 KB) [file 11065_2023_9617_MOESM19_ESM.png]

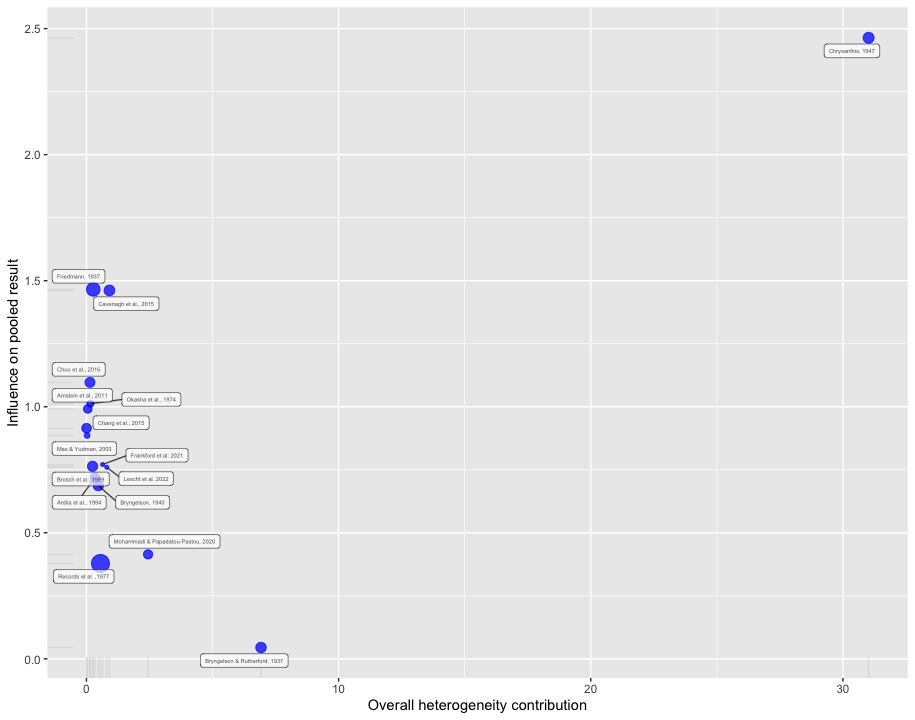

Supplement: Supplementary file 20 — Supplementary file20 (PNG 52 KB) [file 11065_2023_9617_MOESM20_ESM.png]

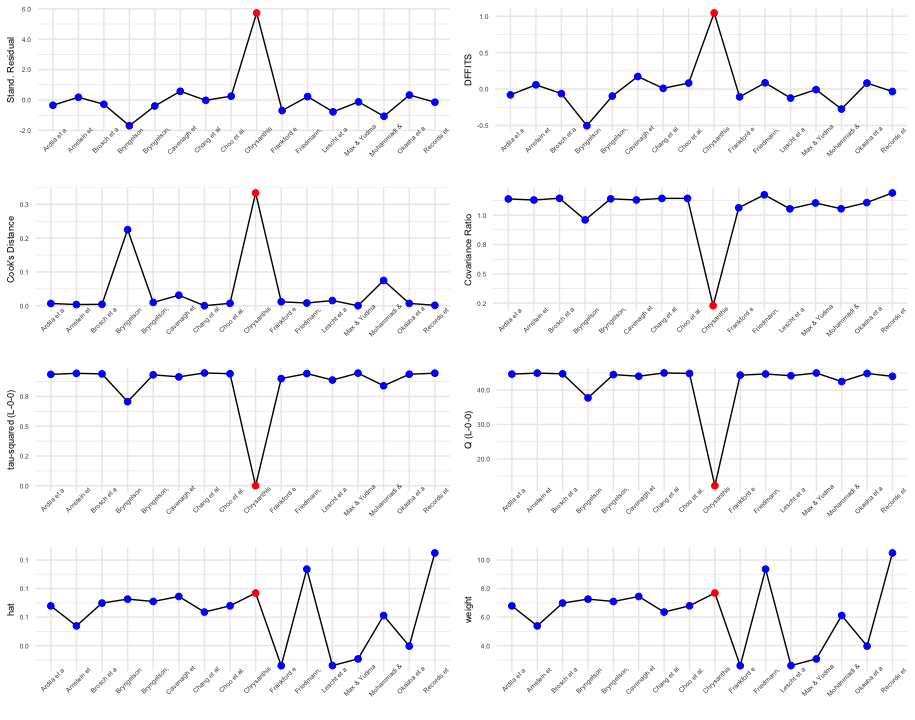

Supplement: Supplementary file 21 — Supplementary file21 (PNG 157 KB) [file 11065_2023_9617_MOESM21_ESM.png]

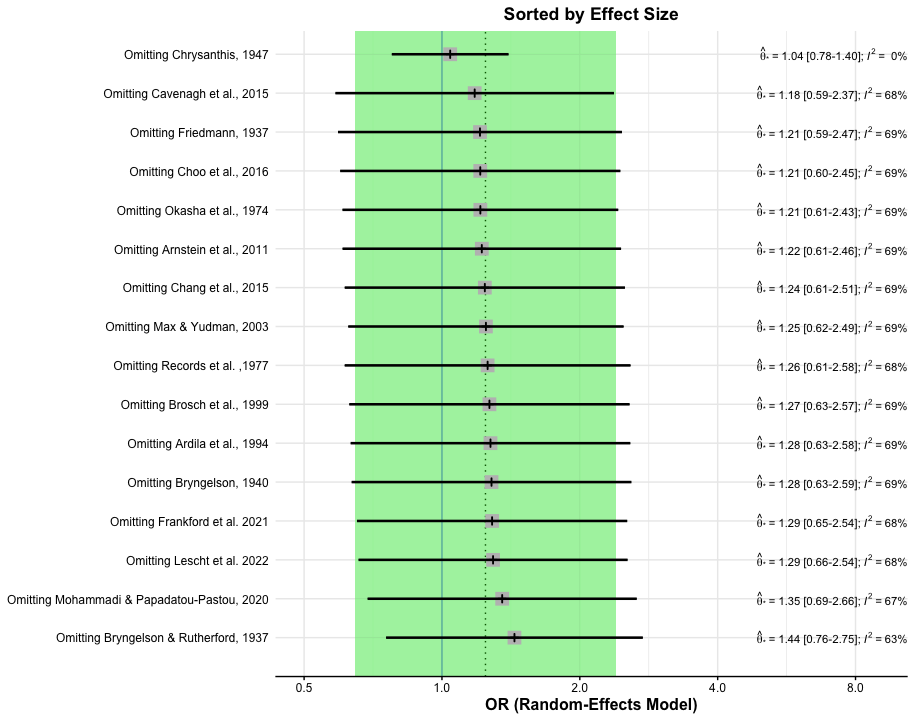

Supplement: Supplementary file 22 — Supplementary file22 (PNG 107 KB) [file 11065_2023_9617_MOESM22_ESM.png]

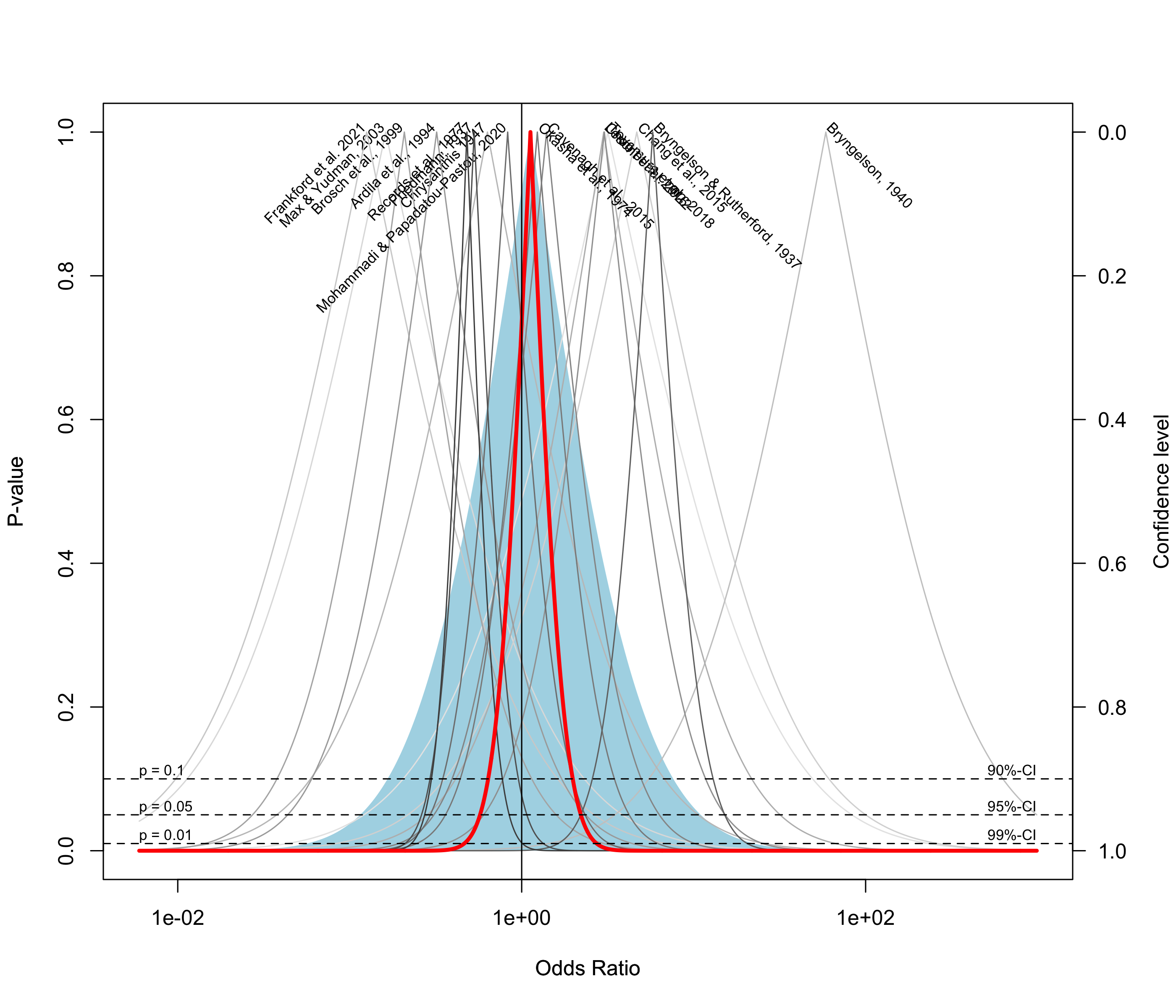

Supplement: Supplementary file 23 — Supplementary file23 (PNG 870 KB) [file 11065_2023_9617_MOESM23_ESM.png]

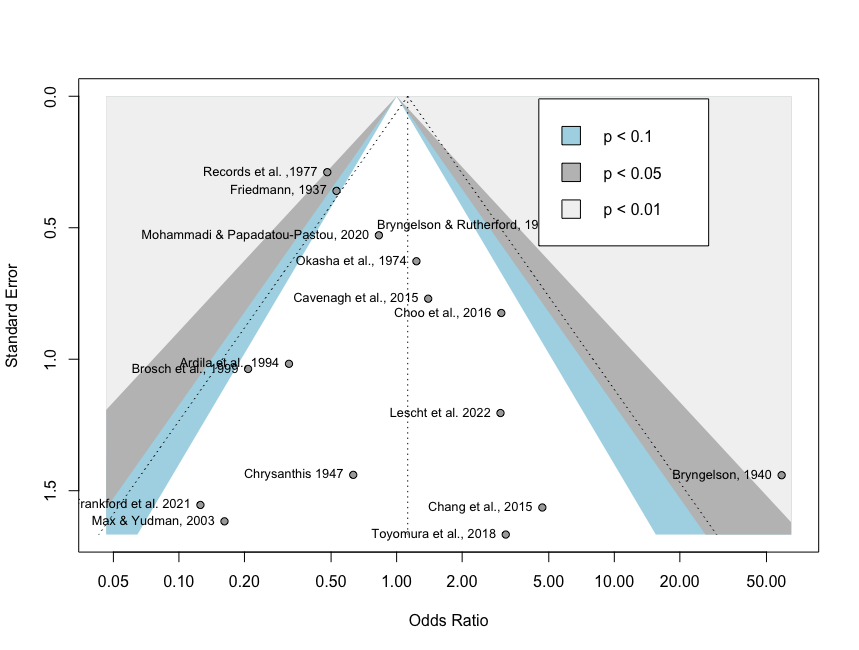

Supplement: Supplementary file 24 — Supplementary file24 (PNG 91 KB) [file 11065_2023_9617_MOESM24_ESM.png]

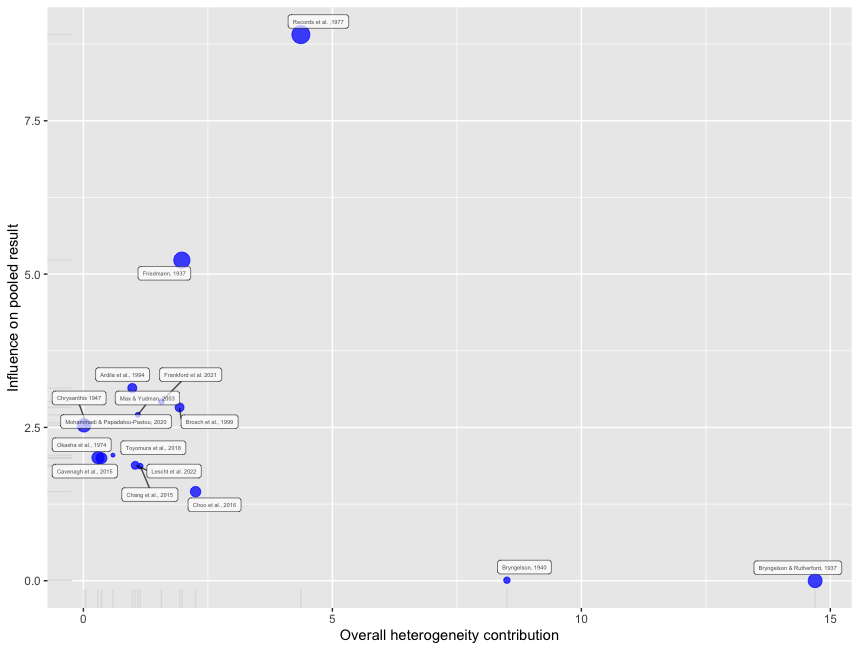

Supplement: Supplementary file 25 — Supplementary file25 (PNG 47 KB) [file 11065_2023_9617_MOESM25_ESM.png]

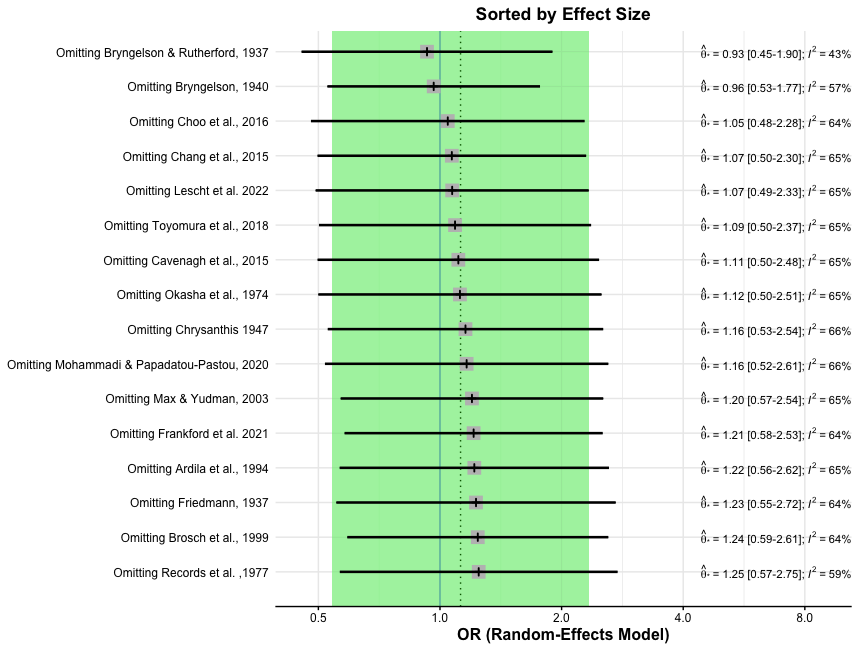

Supplement: Supplementary file 26 — Supplementary file26 (PNG 105 KB) [file 11065_2023_9617_MOESM26_ESM.png]

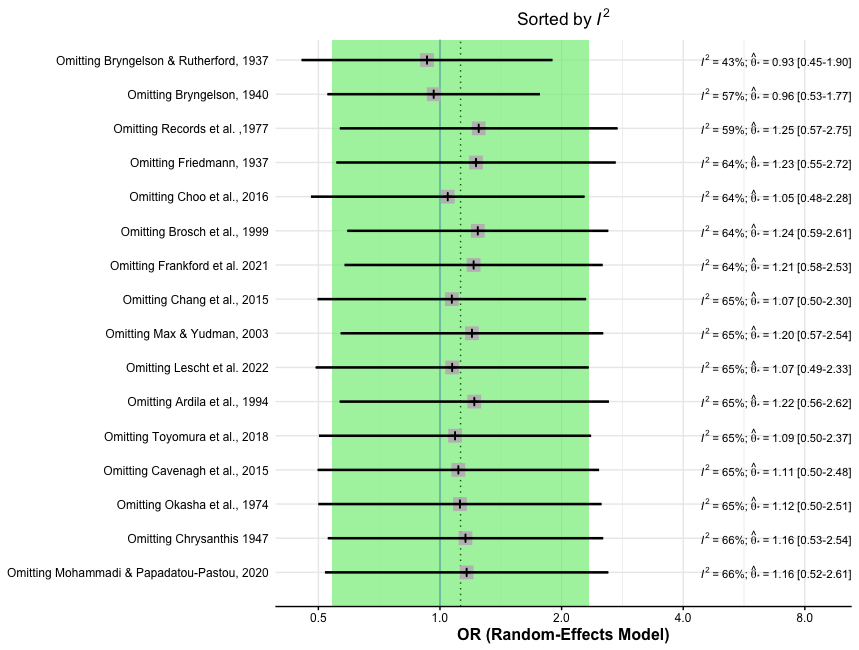

Supplement: Supplementary file 27 — Supplementary file27 (PNG 104 KB) [file 11065_2023_9617_MOESM27_ESM.png]

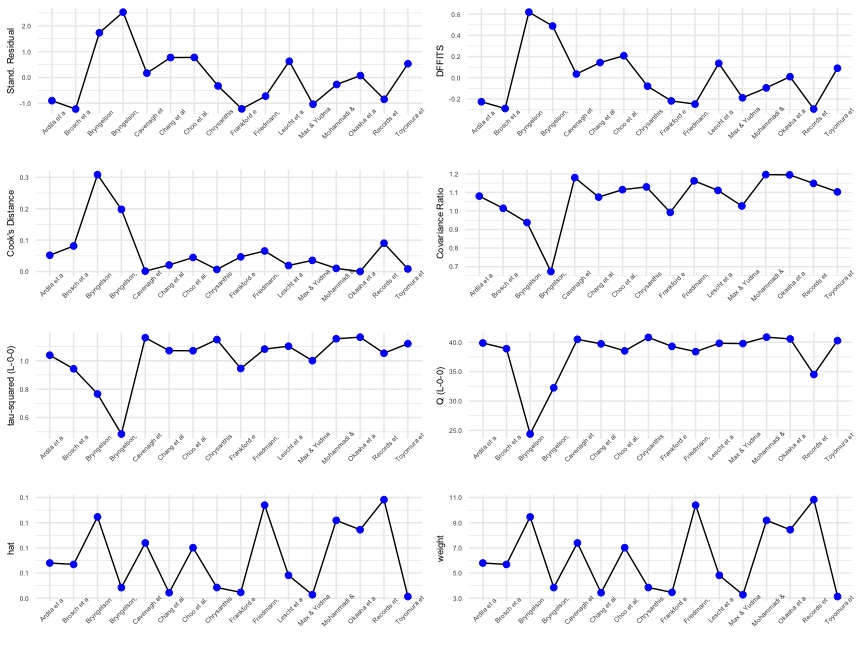

Supplement: Supplementary file 28 — Supplementary file28 (PNG 158 KB) [file 11065_2023_9617_MOESM28_ESM.png]

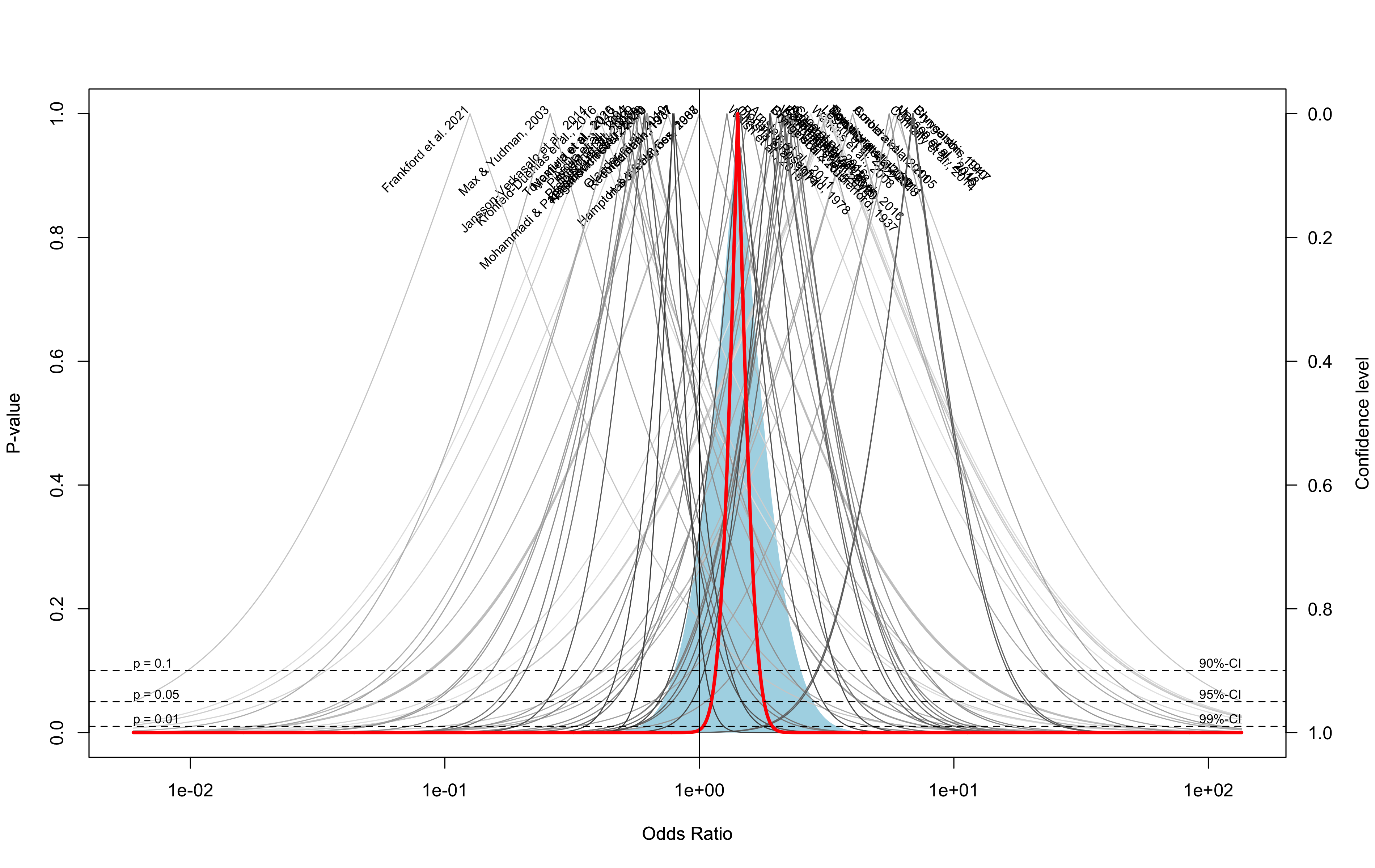

Supplement: Supplementary file 29 — Supplementary file29 (PNG 1616 KB) [file 11065_2023_9617_MOESM29_ESM.png]
